# Transcriptomics reveals *in vivo* efficacy of PARP inhibitor combinatorial synergy with platinum-based chemotherapy in human non-small cell lung carcinoma models

**DOI:** 10.18632/oncotarget.28162

**Published:** 2022-01-03

**Authors:** Lindsay R. Stolzenburg, Barrett Ainsworth, Bridget Riley-Gillis, Tibor Pakozdi, Areej Ammar, Paul A. Ellis, Julie L. Wilsbacher, Cyril Y. Ramathal

**Affiliations:** ^1^AbbVie Inc., North Chicago, IL 60064, USA; ^*^These authors contributed equally to this work

**Keywords:** PARP inhibitor, veliparib, cisplatin, transcriptomics, NSCLC

## Abstract

Inhibitors of poly(ADP)-ribose polymerase (PARP) exploit defective DNA repair pathways existing in several forms of cancer, such as those with BRCA mutations, and have proven clinical efficacy as chemosensitizers. However, platinum-based chemopotentiation by PARP inhibitors (PARPi), particularly for non-small cell lung cancer (NSCLC), has only been confirmed in a few preclinical models and the molecular mechanisms that drive PARPi combinatorial synergy with chemotherapeutics remains poorly defined. To better understand these mechanisms, we characterized cisplatin and veliparib efficacy in A549 and Calu6 NSCLC *in vivo* tumor xenograft models and observed combinatorial synergy in the Calu6 model. Transcriptome-wide analysis of xenografts revealed several differentially expressed genes (DEGs) between untreated and cisplatin + veliparib-treated groups, which were unique from genes identified in either of the single-agent treatment arms. Particularly at 10- and 21-days post-treatment, these DEGs were enriched within pathways involved in DNA damage repair, cell cycle regulation, and senescence. Furthermore, TGF-β- and integrin-related pathways were enriched in the combination treatment arm, while pathways involved in cholesterol metabolism were identified at earlier time points in both the combination and cisplatin-only groups. These data advance the biological underpinnings of PARPi combined with platinum-based chemotherapy and provides additional insight into the diverse sensitivity of NSCLC models.

## INTRODUCTION

Genome instability is a hallmark of most human malignancies and is linked to the initiation and progression of both inherited and sporadic cancers. Defects in DNA repair pathways, often observed in cancer cells, lead to the use of alternative, error-prone repair mechanisms and directly contribute to genome instability and tumorigenesis. For example, cells with mutations in *BRCA1* or *BRCA2* cannot perform DNA double-strand break (DSB) repair by homologous recombination (HR), and accumulate genetic lesions through alternate use of nonhomologous end joining (NHEJ) that directly leads to cancer development [1, 2]. Inhibition of targeted NHEJ and other error-prone repair pathways in BRCA-deficient tumors promotes synthetic lethality and induces cell death [3]. Notably, the development of inhibitors to poly(ADP)-ribose polymerase 1 (PARP1), an enzyme involved in single-strand break (SSB) repair, have illustrated the feasibility and clinical efficacy of this concept [4–6].

The PARP family of enzymes function to transfer ADP-ribose from NAD+ onto target proteins. PARP1, the most abundant family member, recognizes single base pair lesions, binds to DNA, and synthesizes poly(ADP)-ribose (PAR) chains onto nearby protein targets, a function known as PARylation [7]. This action recruits DNA repair machinery to the lesion, particularly effectors involved in base excision repair (BER) such as XRCC1, and eventually leads to PARP1 auto-PARylation and its release from DNA [7, 8]. PARP1 knockout mice exhibit defects in SSB repair but are otherwise viable, only showing subtle impairments in genome stability – likely because PARP1 is generally not involved in HR processes and perhaps due to some functional overlap with PARP2 [8–10]. However, HR-deficient cells lacking BRCA1 or BRCA2 show specific susceptibility to PARP1 inhibition, whereby accumulation of genetic lesions and ensuing chromosome instability in fast-dividing cancer cells results in their death [4, 5]. Similar selective killing by PARP1/2 inhibitors (PARPi) has also been observed in cancer cells with normal *BRCA* alleles, but that molecularly and clinically phenocopy *BRCA* loss – termed “BRCAness” – mediated through mutations in other HR repair genes, such as *ATM, CHEK1*, and *RAD51*, or the tumor suppressor *PTEN* [3, 11].

Growing evidence also indicates that inherent sensitivity to PARPi can be conferred following platinum chemotherapy in cells exhibiting certain gene expression signatures, such as reduced expression of *BRCA1* or *ERCC1* [6, 12, 13]. Preclinical combinations of PARPi with platinating agents have demonstrated synergistic efficacy across diverse tumor classes [14–16]. Mechanistically, PARP1 has been shown to interact with nuclear machinery to coordinate DNA damage repair against platinum-induced DNA damage [17–19] with inhibition of PARP’s DNA repair activity and platinum compound potentiation showing effectiveness in preclinical models. This hypothesis has been tested in clinical trials of platinum-based therapy +/– veliparib in non-small cell lung cancer (NSCLC) and advanced breast and ovarian cancers [20–22]. Mixed clinical outcomes were observed in these trials suggesting that potentiation of PARPi by platinum chemotherapy is context dependent. Despite foundational preclinical work to determine PARP/platinum combinatorial mechanisms in NSCLC, potentiation has only been confirmed in a few NSCLC models, and a clear molecular understanding of the synergy between PARPi and platinum-based chemotherapeutics remains poorly-defined, particularly in *BRCA-*wild type cancers [16, 23].

Transcriptome-wide analysis following treatment with veliparib and cisplatin in NSCLC xenograft models was performed to investigate the differential responses to this combination therapy. While Calu6- or A549-derived xenografts did not regress in response to treatment with cisplatin versus the combination of veliparib and cisplatin, combination therapy-treated Calu6 tumors exhibited significant differential transcriptional changes suggesting that molecular pathways related to DNA damage repair are induced and that pathways involving senescence, TGF-β/WNT signaling, and cholesterol metabolism exhibit pleiotropic effects following combination treatment *in vivo.*

## RESULTS

### Assessment of sensitivity of NSCLC xenograft models to PARPi in combination with cisplatin

**Figure 1:**
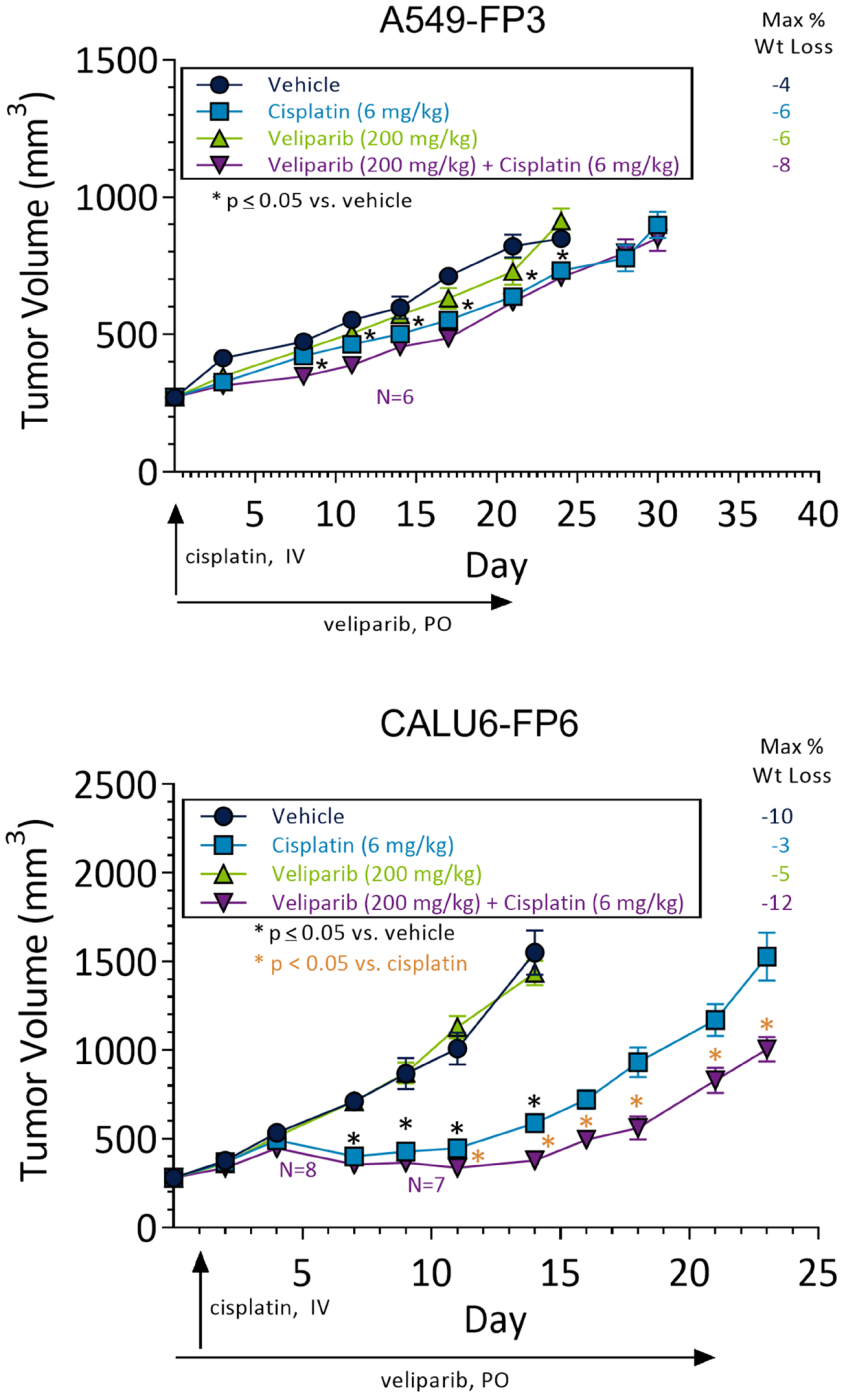
NSCLC xenograft models demonstrate differential sensitivity to cisplatin and veliparib combinations. *In vivo* efficacy in A549-FP3 (*n* = 9) and Calu6-FP6 (*n* = 9) xenografted mice treated with veliparib (200 mg/kg/day BID for 21 days), cisplatin (6 mg/kg IV once) or combinations of both drugs. X-axis shows days since drug treatment start, and drug dosing days are indicated by arrows. ^*^ *p* < 0.05 compared to vehicle control (black) or compared to cisplatin (orange). In combination arms, mice lost due to toxicity are indicated by purple N = X text.

Potentiation of cisplatin by PARP inhibitors has been previously reported in Calu6 and A549 cell lines *in vitro* and *in vivo* [15, 23, 24]. To determine whether *in vivo* models demonstrated increased synergy between cisplatin and veliparib treatment, an efficacy study was performed using A549-FP3 and Calu6-FP6 xenografts implanted in SCID mice. Mice were dosed once with 6 mg/kg cisplatin and/or with vehicle control (VC) or veliparib BID at 200 mg/kg/day for 21 days. A549-FP3 xenografts showed minimal response to single agent cisplatin, single agent veliparib, or combination therapy (Figure 1, top panel). Calu6-FP6 xenografts receiving veliparib alone also demonstrated minimal response when compared to VC (Figure 1, bottom panel). However, significant reduction in tumor volume over VC was observed in cisplatin-treated Calu6-FP6 xenograft mice. Of note, the veliparib and cisplatin combination in Calu6-FP6 xenografts demonstrated additional tumor volume reductions above single-agent cisplatin treated mice, with significant tumor volume reductions becoming apparent at day 10 post-treatment induction and onward. These results support findings in previous studies which suggested synergistic activity between PARPi and cisplatin in *in vivo* models [15, 24].

### Transcriptome-wide changes are observed following cisplatin+veliparib treatment in Calu6-FP6 cells

**Figure 2:**
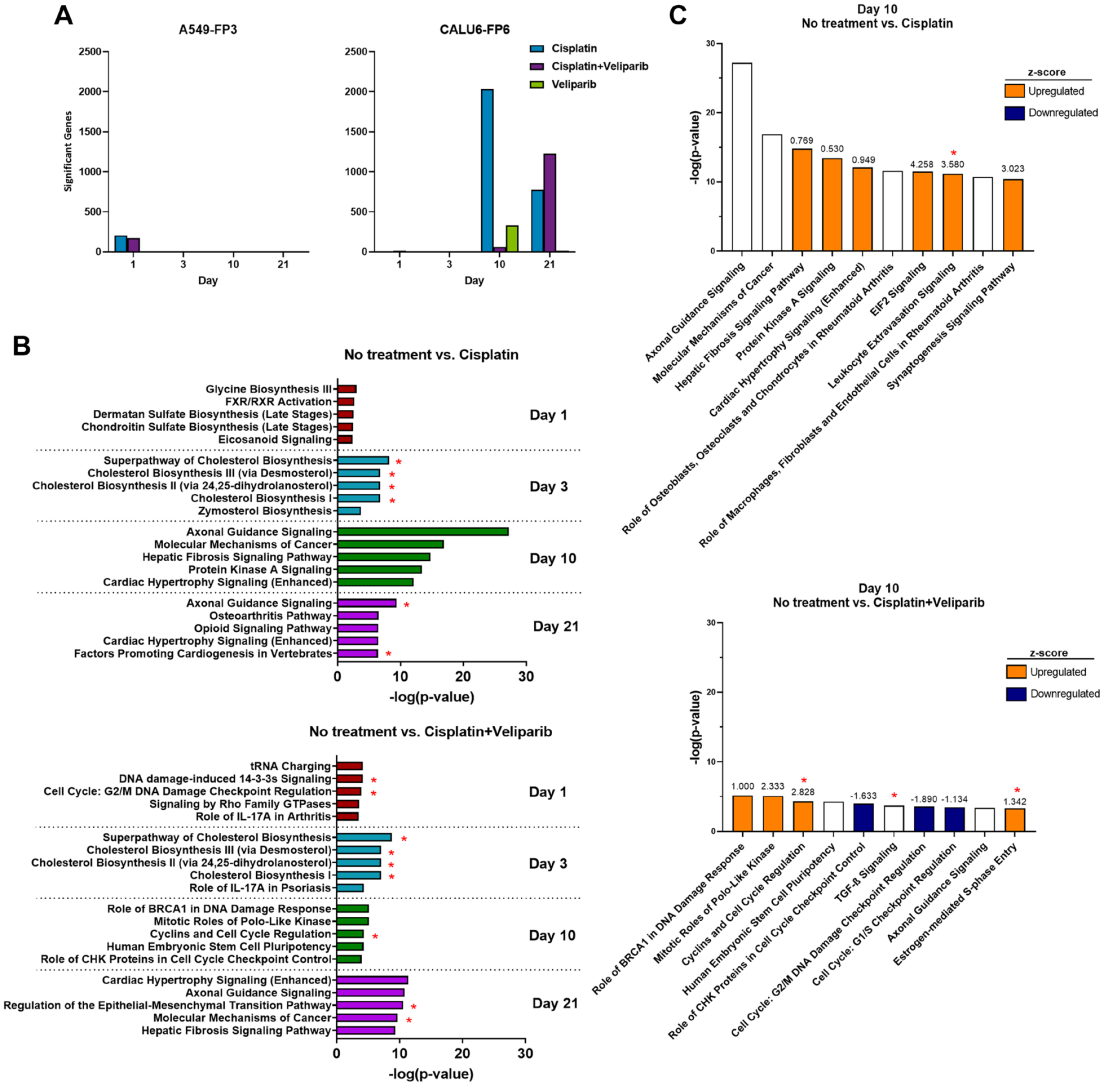
RNA-sequencing and gene ontology enrichment analysis reveal a diverse transcriptional response to cisplatin and veliparib treatment in A549-FP4 and Calu6-FP6 *in vivo* models. (**A**) Temporal transcriptional response to veliparib and cisplatin treatments in A549-FP3 (left) and Calu6-FP6 (right). Significant genes are defined by differential gene expression (DGE) between no treatment and treatment arms at the indicated day with a false-discovery rate of 5% and log2 fold change values greater than 1.0/less than –1.0. (**B**) IPA gene ontology analysis showing top 5 pathways on days 1, 3, 10, and 21 between no treatment vs. cisplatin (top) and no treatment vs. cisplatin + veliparib (bottom). ^*^denotes pathways shared with similar pathways from GSEA at the same time point. (**C**) IPA gene ontology analysis showing top 10 pathways on day 10 between no treatment vs. cisplatin (top) and no treatment vs. cisplatin + veliparib (bottom). Z-scores for pathways are denoted by numbers above each bar and colors: upregulated (orange) and downregulated (navy). ^*^denotes pathways shared with similar pathways from GSEA at the same time point.

To understand the molecular mechanisms by which potentiation occurs *in vivo*, we performed a transcriptome-wide analysis. Growth of A549-FP3 and Calu6-FP6 xenografts was measured over time in mice treated once with 4.5 mg/kg cisplatin, 200 mg/kg/day veliparib QD x 21 days, or the combination (Supplementary Figure 1). Tumors were harvested at days 1, 3, 10 and 21 after cisplatin dosing from 5 mice per time point and total RNA was extracted for RNA sequencing (RNA-seq) library preparation and sequencing, and differential gene expression analysis. The number of significant differentially expressed genes (DEGs) was determined between treatment arms and VC using a false discovery rate of 0.05 and log_2_ fold change values greater than 1.0 and less than –1.0 (Figure 2A).

In A549-FP3 xenografts, similar numbers of DEGs were observed between cisplatin compared to VC, and cisplatin + veliparib compared to VC, at day 1 (205 and 173, respectively). Zero genes were significantly different in the veliparib-only treatment arm at this time point compared to VC, and no DEGs were identified across all conditions at the later time points. In contrast, Calu6-FP6 xenografts displayed a diverse transcriptional response, with 5, 9, 2033, and 775 DEGs identified between cisplatin and VC at days 1, 3, 10, and 21, respectively, and 15, 12, 61, and 1228 DEGs observed across the same time points in the combination cisplatin + veliparib treatment arm. Single agent veliparib resulted in 332 DEGs compared to VC at day 10, but only 13 genes spread across the three other time points. Particularly at days 10 and 21, many DEGs in Calu6-FP6 cells are shared between the cisplatin-only and cisplatin + veliparib treatment arms (42 and 601, respectively), but the majority of genes are unique to cisplatin alone on day 10 (1704) and cisplatin + veliparib on day 21 (626) (Supplementary Figure 2). Notably, *PARP1* and *PARP2* expression were largely unchanged across time and between treatment arms (Supplementary Figure 3). These results highlight the broad transcriptome-level effects that cisplatin exerts in Calu6-FP6 cells and indicates the potential for combination veliparib to alter additional genes and pathways compared to cisplatin alone.

To assess biological pathways that may be dysregulated in response to single agent cisplatin compared to combination cisplatin + veliparib therapy, we next performed pathway analysis on Calu6-FP6 DEGs using the Ingenuity Pathway Analysis tool (Figure 2B). Compared to VC, cisplatin treatment alone altered genes in pathways related to cholesterol and sugar biosynthetic processes at days 1 and 3, and broad, nondescript pathways at days 10 and 21, including axonal guidance signaling, molecular mechanisms of cancer, and cardiac hypertrophy signaling. Several of these pathways were also found using the Gene Set Enrichment Analysis tool (GSEA, [25, 26]), particularly the cholesterol biosynthesis pathways at day 3 (Supplementary Figure 4A; shared pathways denoted by ^*^). GSEA also identified additional gene sets of interest, including upregulated immune-related gene sets at days 10 and 21, and downregulated gene sets involved in mitochondrial activity. On the other hand, top pathways identified by IPA in Calu6-FP6 treated with combination cisplatin + veliparib included DNA damage at day 1, DNA repair and cell cycle control at day 10, and epithelial-mesenchymal transition (EMT) regulation at day 21 (Figure 2B). Particularly at day 10, many of the DNA repair, cell cycle-related, and EMT pathways were shared with enriched gene sets from GSEA (Figure 2C, Supplementary Figure 4B). The direction (upregulated vs. downregulated) of these pathways was mixed, however. Interestingly, cholesterol biosynthesis pathways were also overrepresented at day 3, similar to results observed with cisplatin alone. Overall, these results highlight the divergent transcriptional responses of A549-FP3 and Calu6-FP6 to cisplatin and veliparib and demonstrate the synergistic effects of combination cisplatin + veliparib treatment on genes involved in DNA repair, cell cycle, and EMT in Calu6-FP6, which were not observed in this line with cisplatin alone or in A549-FP3 cells.

### Pathways and genes related to DNA repair and cell cycle regulation are altered in Calu6-FP6 cells treated with cisplatin and veliparib

**Figure 3:**
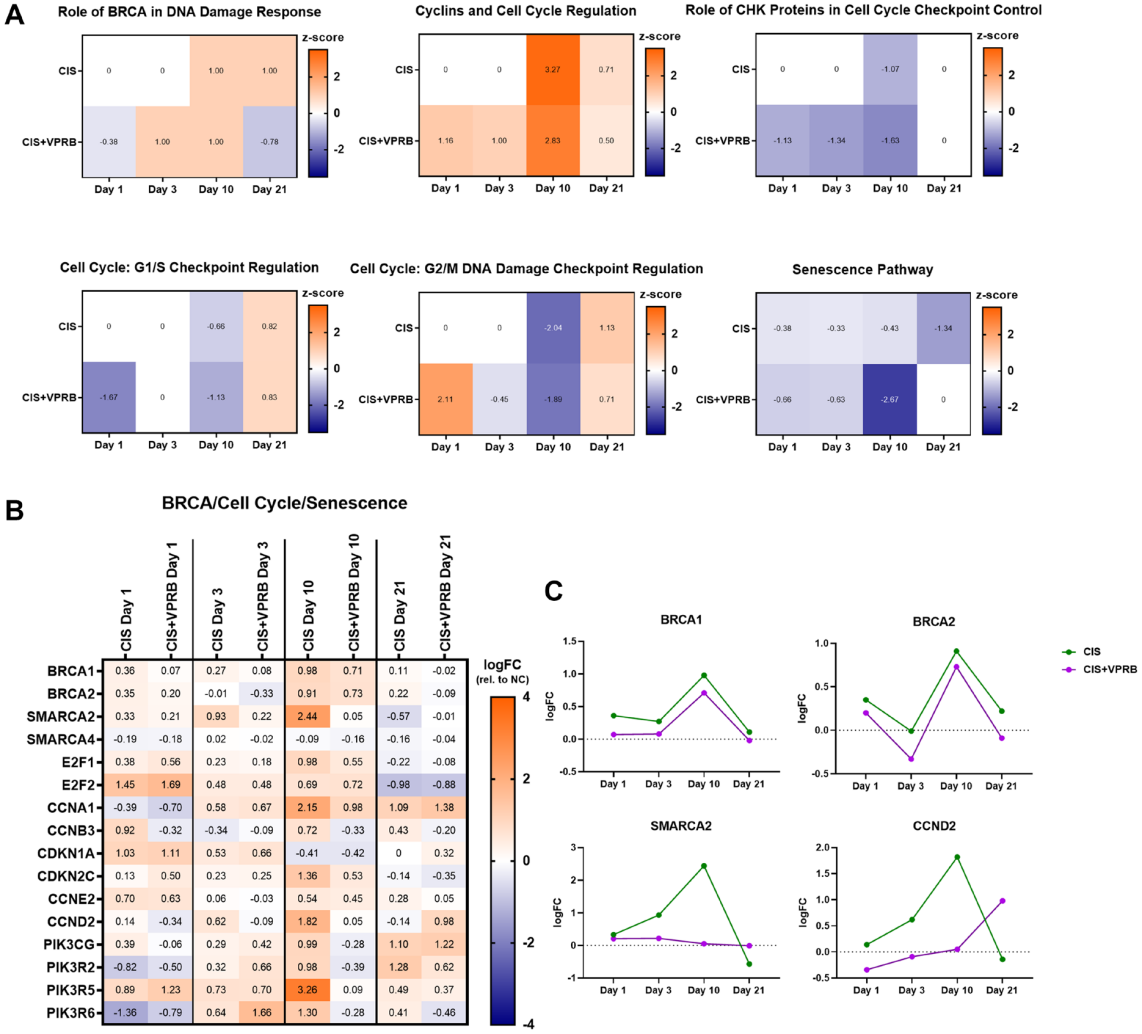
Cell cycle and DNA repair pathways in Calu6-FP6 models treated with cisplatin and veliparib. (**A**) Heatmaps showing IPA z-score values for selected pathways in no treatment vs. cisplatin (CIS) and no treatment vs. cisplatin + veliparib (CIS+VPRB) on days 1, 3, 10, and 21. (**B**) Heatmap illustrating log fold change (logFC) values for selected genes within cell cycle and DNA pathways of interest. (**C**) Log fold change (logFC) plots for BRCA1, BRCA2, SMARCA2, and CCND2.

Our initial IPA and GSEA analysis revealed enrichment of several pathways related to DNA damage response and cell cycle control in Calu6-FP6 cells treated with cisplatin + veliparib, so we next performed a more comprehensive review of these pathways and the genes contained within them. We focused on day 10, since this time point appeared to produce the most divergent number of DEGs and pathways between treatment arms. Top pathways in cisplatin + veliparib compared to VC included Role of BRCA in DNA Damage Response, Cyclins and Cell Cycle Regulation, Role of CHK Proteins in Cell Cycle Checkpoint Control, Cell Cycle: G2/M DNA Damage Checkpoint Regulation, Cell Cycle:G1/S Checkpoint Regulation, and Senescence Pathway (see Supplementary Table 1 for -log(*p*-values)). *Z*-scores indicated that Role of BRCA in DNA Damage Response and Cyclins and Cell Cycle Regulation were upregulated at day 10, while the remaining 4 pathways were downregulated (Figure 3A), suggesting increased DNA damage responses and decreased cell cycling and proliferative capacity in response to cisplatin and veliparib. However, z-scores from the same pathways in the cisplatin-only treatment arm were relatively comparable across time points, albeit the level of significance observed for each pathway at day 10 was more mixed (Supplementary Table 1). A notable exception to this was the Senescence Pathway, which was strongly downregulated at day 10 following cisplatin + veliparib (–2.67) compared to cisplatin treatment alone (–0.43).

Though pathway enrichment scores were largely shared between the two treatment arms, we nevertheless observed several genes within these pathways as having divergent expression, particularly at day 10 (Figure 3B). Contained within several of the cell cycle regulation pathways, Cyclin B3 (*CCNB3*), for example, is decreased following cisplatin + veliparib treatment at day 10 and 21, while being upregulated at the same time points after single-agent cisplatin. Similarly, Cyclin D2 (*CCND2*) was also increased following cisplatin treatment, but not with cisplatin + veliparib (Figure 3B and 3C). The global transcription factor *SMARCA2*, contained within the Role of BRCA in DNA Damage Response Pathway and important for increasing accessibility to damaged chromatin, was also strongly upregulated in the cisplatin-only treatment arm, but unchanged following cisplatin + veliparib treatment (Figure 3B, 3C). Notably, *BRCA1* and *BRCA2* levels were both slightly upregulated at day 10, but their expression did not differ dramatically between treatment arms (Figure 3C). Finally, we found several members of the phosphoinositide 3-kinase (PI3K) family, contained within the Senescence Pathway and involved in several cellular processes related to cell signaling and proliferation, as also strongly upregulated following cisplatin treatment, but not with cisplatin + veliparib (Figure 3B). Although we detected the majority of Calu6-FP6 DEGs to cluster mostly by time point rather than treatment arm, a number of genes important for cell cycle control and survival were decreased following cisplatin + veliparib treatment, suggesting the greater impact combination treatment may have on these cellular processes compared to cisplatin treatment alone.

### Additional pathways and genes related to EMT and cholesterol biosynthesis are altered in Calu6-FP6 xenografts treated with cisplatin and veliparib

**Figure 4:**
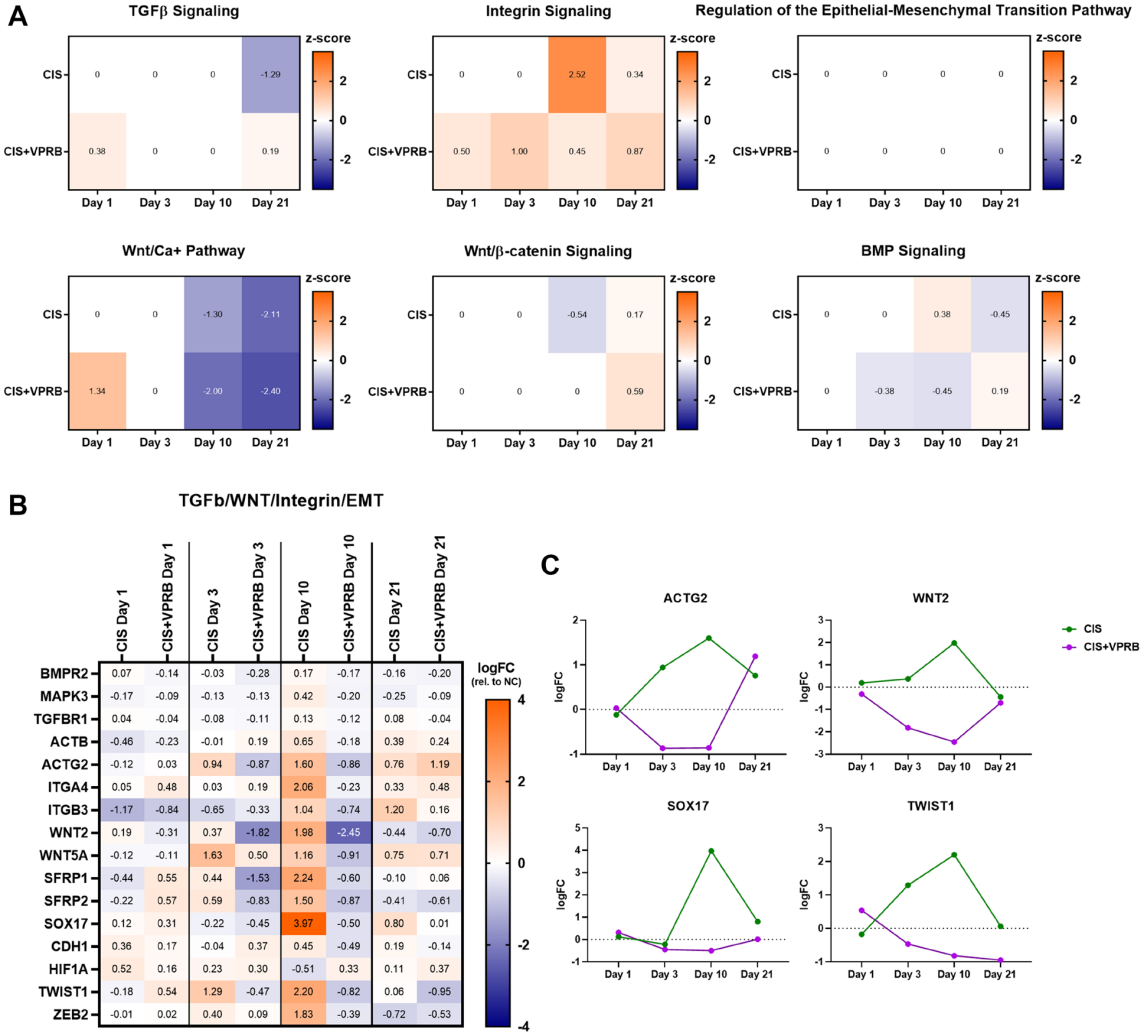
TGF-b, WNT, and integrin signaling pathways in Calu6-FP6 models treated with cisplatin and veliparib. (**A**) Heatmaps showing IPA z-score values for selected pathways in no treatment vs. cisplatin (CIS) and no treatment vs. cisplatin + veliparib (CIS+VPRB) on days 1, 3, 10, and 21. (**B**) Heatmap illustrating log fold change (logFC) values for selected genes within TGFβ, WNT, and integrin signaling pathways of interest. (**C**) Log fold change (logFC) plots for ACTG2, WNT2, SOX17 and TWIST1.

The transforming growth factor beta (TGF-β) Signaling Pathway was one of the top IPA/GSEA pathways enriched in Calu6-FP6 cells treated with cisplatin + veliparib at day 10, and the associated Regulation of the Epithelial-Mesenchymal Transition Pathway was enriched at day 21 (Figure 2 and Supplementary Figure 4). Given the importance these pathways play in cancer progression, we next probed these and other related pathways further (Integrin Signaling, Wnt/Ca+ Pathway, Wnt/β-catenin Signaling, and BMP Signaling) to identify DEGs within them that may drive this signal. Closer analysis of day 10 IPA data revealed that, although TGF-β Signaling is a top pathway in the cisplatin + veliparib treatment group, the pathway is statistically more enriched following cisplatin treatment alone (Supplementary Table 2); similarly, the other related pathways also have higher levels of significance with single-agent cisplatin than in the combination treatment. Z-scores for most pathways are near-zero, or do not show meaningfully different directions of dysregulation between the two treatment arms, apart from TGF-β Signaling at day 21, which is downregulated with cisplatin treatment alone and slightly upregulated with cisplatin + veliparib, and Integrin Signaling at day 10, which is highly upregulated following cisplatin treatment alone and only marginally so with combination treatment (Figure 4A). Nevertheless, many DEGs contained within these pathways trend in opposite directions between the different treatment groups at day 10 (Figure 4B). For example, members of the actin (*ACTB*, *ACTG2*) and integrin (*ITGA4*, *ITGB3*) families are strongly increased with single agent cisplatin, and moderately decreased with combination therapy (Figure 4B and 4C). *WNT2* and *ZEB2*, both implicated in cancer progression, and *CDH1* and *SOX17*, having the opposite function in cell fate determination and tumor suppression, all also follow the abovementioned expression pattern at multiple time points (Figure 4B and 4C). These data likely account for the neutral z-scores we observed for most of these pathways and suggest that the effects of cisplatin + veliparib can generate multiple cellular responses that may simultaneously increase and decrease cancer progression phenotypes.

**Figure 5:**
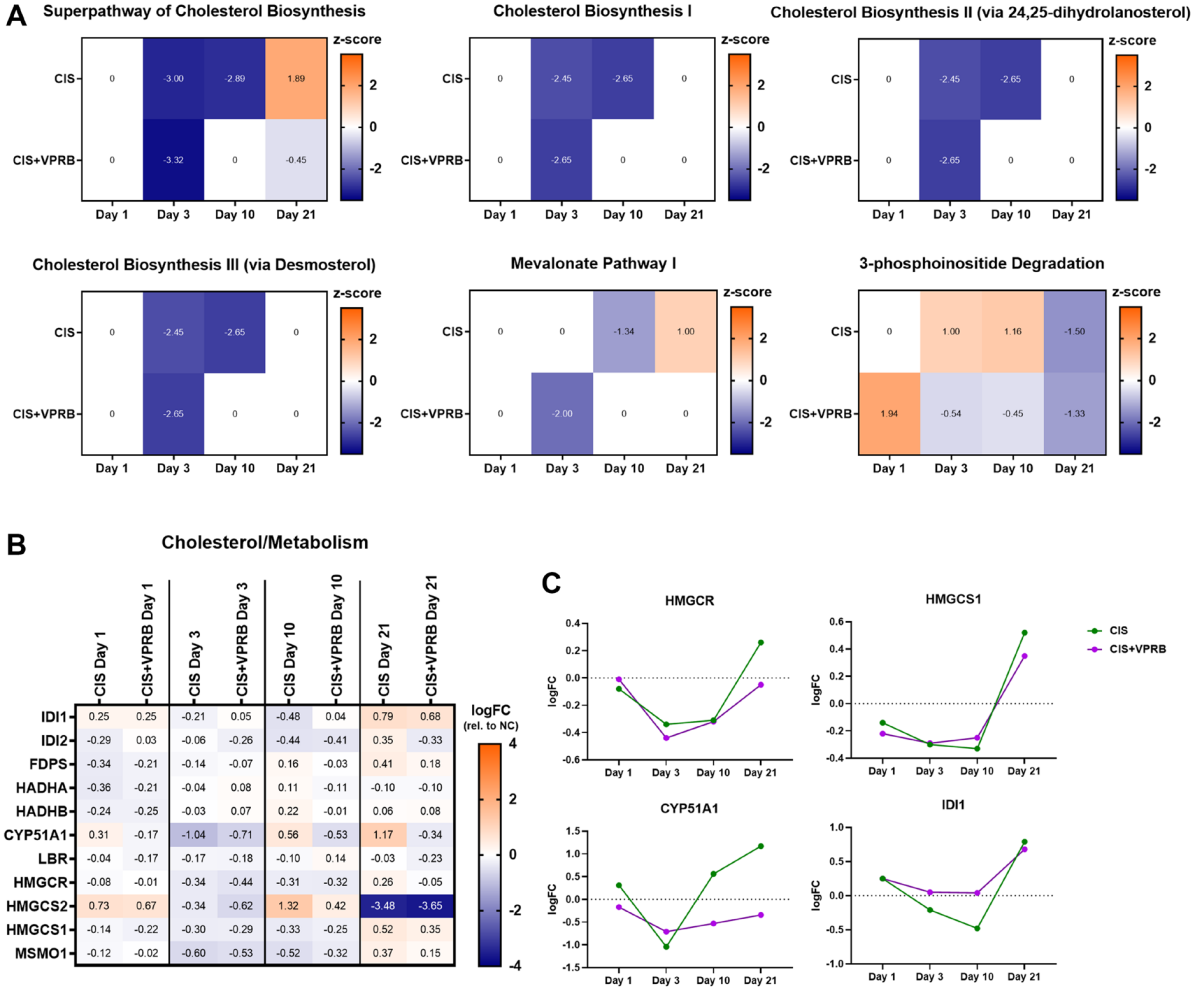
Cholesterol biosynthesis pathways in Calu6-FP6 models treated with cisplatin and veliparib. (**A**) Heatmaps showing IPA z-score values for selected pathways in no treatment vs. cisplatin (CIS) and no treatment vs. cisplatin + veliparib (CIS+VPRB) on days 1, 3, 10, and 21. (**B**) Heatmap illustrating log fold change (logFC) values for selected genes within cholesterol biosynthesis pathways of interest. (**C**) Log fold change (logFC) plots for HMGCR, HMGCS1, CYP51A1, and IDI1.

Our RNA-seq analysis also revealed significant enrichment of several pathways related to cholesterol biosynthesis, both in xenografts treated with single agent cisplatin and the combination therapy at day 3 (Supplementary Table 3, Figure 2B and Supplementary Figure 4). Cholesterol metabolism, specifically targets regulated by the master transcription factor SREBP2, is increasingly recognized to be upregulated during tumorigenesis, likely because continued cell growth is dependent on lipid production [27, 28]. Compared to VC, we observed strong downregulation of four pathways related to cholesterol biosynthesis in both treatment arms at day 3 (Superpathway of Cholesterol Biosynthesis, Cholesterol Biosynthesis I, Cholesterol Biosynthesis II, and Cholesterol Biosynthesis III) (Figure 5A). Mevalonate Pathway I, providing a crucial precursor for cholesterol biosynthesis, shows a similar trend. These results suggest diminished dependence on lipid production by Calu6-FP6 cells due to chemotherapy-induced decreases in proliferation. Interestingly, cholesterol biosynthesis downregulation is sustained through day 10 in the cisplatin-only treatment arm, while becoming neutral in cisplatin + veliparib. Further inspection of DEGs contained within these pathways revealed similar trends of downregulation in both treatment arms compared to VC at day 3 (Figure 5B). More specifically, members of the mevalonate pathway, including 3-hydroxy-3-methylglutaryl-CoA (HMG-CoA) reductase (*HMGCR*, the target for statins), HMG-CoA synthase (*HMGCS1* and *HMGCS2*), and isopentenyl pyrophosphate isomerase (*IDI1* and *IDI2*), were all downregulated at day 3 (Figure 5B and 5C). The lanosterol 14-alpha demethylase *CYP51A1* was also decreased at day 3, though its expression became more divergent between the two treatment arms by day 10. Interestingly, we also identified the 3-phosphoinositide Degradation pathway as upregulated following cisplatin treatment alone at days 3 and 10, but downregulated with cisplatin + veliparib (Figure 5A); in addition to other cellular signaling processes, this pathway is also involved in cholesterol biosynthesis [29] and may be driven by dysregulation of PIK3 genes previously discussed as part of the Senescence Pathway (Figure 3).

## DISCUSSION

Despite major progress in understanding platinum adduct repair processing, the mechanism(s) underlying the efficacy of PARP inhibitor potentiation of platinum-based chemotherapy combinations remains elusive. To better understand biological mechanisms that drive PARPi synergism with chemotherapy, we used *in vivo* xenograft tumor models to explore the molecular mechanisms of combinatorial synergy. Similar to previous reports [24], *in vivo* synergy of veliparib with cisplatin therapy was weak. However, by RNA-seq we identified differential gene expression between the combination treatment arm compared to cisplatin alone in Calu6-FP6 tumors. Gene expression differences on day 10 and 21 were particularly notable and coincided with the smallest tumor volumes observed *in vivo* across all treatment arms.

We found that DNA damage repair molecular machinery was similarly activated in the veliparib + cisplatin treatment arm compared to tumors treated with cisplatin alone. Specifically on day 10, DNA damage checkpoint pathways such as 14-3-3, polo-like kinase and BRCA1-dependent repair machinery, were equally activated in both Calu6-FP6 treatment groups. However, specific genes related to DNA damage/repair and cell cycle, including *SMARCA2* and *CCND2*, were strongly upregulated in the cisplatin-only treatment arm at day 10. *SMARCA2* and its paralog, *SMARCA4,* are part of the SWI/SNF complex, which functions to increase chromatin accessibility around double-stranded breaks to promote an efficient repair response [30, 31]. Our observed increases in *SMARCA2* expression from day 1 to day 10 in the cisplatin-only treatment arm may signal a mounting DNA repair response during this time period in the cisplatin-only arm, which is prevented by the addition of veliparib. These data suggest that genes such as *SMARCA2* be further analyzed in the larger context of DNA repair biomarkers that could be utilized as predictors of PARPi efficacy [18].

Similar to the effects we observed for DNA repair pathways, pathways related to TGF-β, WNT, and Integrin signaling were similarly enriched in Calu6-FP6 xenografts treated with cisplatin alone or cisplatin + veliparib. However, several genes within these pathways, including *WNT2, SOX17, TWIST1*, and *ZEB1,* were upregulated by cisplatin alone and downregulated by combination therapy at day 10. Many of these genes/pathways contribute to the process of epithelial-to-mesenchymal transition (EMT), marked by loss of E-cadherin (*CDH1*) expression. Indeed, we find decreased levels of *CDH1* at day 10 following combination therapy in Calu6-FP6 cells, suggesting a transition to an EMT-like phenotype. PARP1 has been found to induce EMT in NSCLC cells via direct transcriptional downregulation of the ZEB1 and Snail transcription factors [32, 33]. Previous studies have also indicated the correlation of EMT phenotypes with cisplatin resistance [34], and shown that cisplatin-resistant NSCLC lines have increased markers of EMT [35]. Confounding this theory is our finding of decreased expression of *SOX17*, an inhibitor of EMT [36], at day 10 in the cisplatin + veliparib arm. Together, we believe these mixed gene-effects account for the neutral z-scores of EMT-related pathways obtained from IPA.

The senescence pathway was negatively regulated across all days and treatment arms in this study, with the strongest negative z-score at day 10 in the cisplatin + veliparib arm. Chemotherapeutics are known inducers of senescence, as are PARP inhibitors [37]. However, the combinatorial synergy of cisplatin + veliparib appears to manifest in Calu6-FP6 as conferring decreased senescence at the transcriptional level compared to cisplatin treatment alone, even though tumor volume was reduced. Contained within the senescence pathway are a number of the *PIK3* genes that comprise a family of lipid kinases that regulate a diverse range of cell functions related to proliferation and survival [38–40]. Notably, pathways involved in cholesterol metabolism and 3-phosphoinositide degradation also showed divergent z-scores between treatment arms at day 10, with cholesterol metabolism being less downregulated and 3-phosphoinositide degradation being more downregulated with cisplatin + veliparib compared to cisplatin alone. PI3K signaling is a known activator of cholesterol synthesis, which is necessary for cell cycling, cell growth, and can contribute to cancer aggressiveness via signaling through the PI3K-AKT-mTOR axis [41–43]. One important gene involved in cholesterol catalysis from lanosterol, CYP51A1 [37], is notably downregulated in expression at day 10 in veliparib + cisplatin treated xenografts compared to those treated with cisplatin alone, suggesting that cholesterol biosynthesis may be reduced in these tumors. Moreover, activated phosphoinositide pathway members in the cisplatin-only treated tumors is further suggestive of increased PI3K-AKT signaling leading to tumor metastasis and tumor growth. Collectively, these findings may suggest that PARPi may block the convergence of PI3K-AKT signaling and cholesterol production.

Taken together, these results highlight several biological pathways and genes that are differentially regulated following Calu6-FP6 xenograft treatment with combination cisplatin and veliparib compared to cisplatin treatment alone, and point towards different avenues for further exploration and target identification.

## MATERIALS AND METHODS

### Cell lines and reagents

Cell lines were provided by AbbVie’s Biobank and were authenticated by STR analysis. A549-flank passage x 3 (FP3) and Calu6- flank passage x 6 (FP6) cell lines were generated after 3 passages of A549 (ATCC) and 6 passages of Calu-6 (ATCC) in the flank of female SCID mice. Prior to inoculation into mice, cell lines were maintained in DMEM with 10% heat-inactivated FBS at 5% CO_2_ and 37°C.

### Xenograft tumor preparation and *in vivo* pharmacology

C.B-17 SCID female mice were obtained from Charles River (Wilmington, MA) and used for all studies. A total of 2 × 10^6^ viable A549-FP3 or Calu6-FP6 cells were inoculated subcutaneously into the right flank of female C.B-17 SCID mice on Day 0. The injection volume was 0.1 mL and was composed of a 1:1 mixture of S-MEM and matrigel. Tumors were size matched at ~250 mm^3^. Veliparib was synthesized at AbbVie (Abbott Park, IL) and formulated in 0.9% saline. Cisplatin was manufactured by Teva Parenteral Medicines, Inc. (Irvine, CA, USA) and was formulated in 0.9% saline. Cisplatin was dosed IV once at 6 mg/kg for preliminary efficacy experiments and at 4.5 mg/kg for the RNA-seq/concurrent efficacy study, while vehicle or veliparib were dosed orally at 200 mg/kg/day twice a day (BID) × 21 days for the preliminary efficacy study and once a day (QD) at 200 mg/kg/day × 21 days for the RNA-seq/concurrent efficacy study.

Tumor volume was calculated two times weekly for A549-FP3 efficacy studies and three times weekly for Calu6-FP6 efficacy studies and on the day of tumor harvest for RNA-seq studies. Tumor samples for the RNA-seq studies were collected at 24 hr, 48 hr, 72 hr, 10 days, and 21 days after cisplatin dosing. Mice were euthanized on the assigned harvest day for the RNA-seq studies or when tumor volume was ≤ 3000 mm^3^ or skin ulcerations occurred for efficacy studies.

### Tumor harvest and nucleic acid isolation

Xenograft tumors were harvested at the indicated times and immediately prepared for dissociation using the Tumor Dissociation Kit (Miltenyi Biotech), according to the manufacturer’s instructions. Briefly, tumors were dissected into ~1 g segments and heated in enzyme mix on the gentleMACS Octo Dissociator. Dissociated cells were strained through a 70 μm MACS SmartStrainer and pelleted to remove debris. Erythrocytes and dead cells were removed by incubation in Red Blood Cell Lysis Solution, and mouse cells were removed with the Mouse Cell Depletion Kit (Miltenyi Biotech), according to manufacturer’s protocol. Purified human cells were immediately processed for RNA and DNA isolation using the AllPrep DNA/RNA Isolation Kit (Qiagen) according to the manufacturer’s instructions.

### RNA-seq prep

For each timepoint, RNA from five xenografts was used per treatment group. RNA library preparation from total RNA was conducted following the manufacturer’s protocol for the Illumina Truseq Stranded Library Prep Gold with Ribo-Zero rRNA Removal Kit. Briefly, 200 ng – 1000 ng of total RNA was purified by Ribo-Zero to remove rRNA and fragmented by divalent cations under elevated temperature. The fragmented RNA underwent first strand synthesis using reverse transcriptase and random primers. Second strand synthesis created the cDNA fragments using DNA polymerase I and RNaseH. The cDNA fragments were end repaired, 3’ ends adenylated, and adapters ligated. The cDNA library was enriched using 10 cycles of PCR and purified. Final libraries were assessed using the Agilent Tapestation and Qubit (ThermoFisher) assay methods and sequenced on an Illumina HiSeq 3000 sequencer using 2 × 75 bp read length and 20–40 M reads per sample.

### RNA-seq analysis

Sequencing reads were aligned to the hg38 human genome with STAR [44] under default parameters. Failed, multi-mapped, and PCR-duplicated reads were removed using bash scripting and samtools, and quality control was performed using FastQC, PICARD tools, Qualimap, and Samtools-stats on MultiQC software (Supplementary Table 4). Biological QC was performed using customized R scripts. Alignment files were summarized into count matrices using subread featureCounts. For differential gene expression analysis, count matrices were selected in parallel for protein-coding genes or filtered for counts-per-million of 1 (CPM) average value across samples. Data was normalized using the TMM approach and mean-variance relationship was estimated with the Limma-VOOM algorithm. The values of VOOM fitting were then used as priors to the empirical bayes moderated t-statistics.

### Pathway and gene set enrichment analysis

Results from RNA-seq studies were analyzed using Ingenuity Pathway Analysis (IPA, Qiagen Inc., https://www.qiagenbioinformatics.com/products/ingenuitypathway-analysis) using the core analysis function and an FDR *q*-value cutoff of 0.2. Gene Set Enrichment Analysis (GSEA, [25, 26]) was performed using Hallmark, C2, C5, and C6 gene sets and an FDR *q*-value cutoff of 0.2. Venn diagrams were generated using http://bioinformatics.psb.ugent.be/webtools/Venn/.

## SUPPLEMENTARY MATERIALS





## Abbreviations

PARP: poly(ADP)-ribose polymerase
PARPi: poly(ADP)-ribose polymerase inhibitors
NSCLC: non-small cell lung cancer
DEGs: differentially expressed genes

## References

[1] Venkitaraman AR . Cancer suppression by the chromosome custodians, BRCA1 and BRCA2. Science. 2014; 343:1470–75. 10.1126/science.1252230. 24675954

[2] Tutt A , Bertwistle D , Valentine J , Gabriel A , Swift S , Ross G , Griffin C , Thacker J , Ashworth A . Mutation in Brca2 stimulates error-prone homology-directed repair of DNA double-strand breaks occurring between repeated sequences. EMBO J. 2001; 20:4704–16. 10.1093/emboj/20.17.4704. 11532935 PMC125603

[3] Lord CJ , Tutt AN , Ashworth A . Synthetic lethality and cancer therapy: lessons learned from the development of PARP inhibitors. Annu Rev Med. 2015; 66:455–70. 10.1146/annurev-med-050913-022545. 25341009

[4] Farmer H , McCabe N , Lord CJ , Tutt AN , Johnson DA , Richardson TB , Santarosa M , Dillon KJ , Hickson I , Knights C , Martin NM , Jackson SP , Smith GC , Ashworth A . Targeting the DNA repair defect in BRCA mutant cells as a therapeutic strategy. Nature. 2005; 434:917–21. 10.1038/nature03445. 15829967

[5] Bryant HE , Schultz N , Thomas HD , Parker KM , Flower D , Lopez E , Kyle S , Meuth M , Curtin NJ , Helleday T . Specific killing of BRCA2-deficient tumours with inhibitors of poly(ADP-ribose) polymerase. Nature. 2005; 434:913–17. 10.1038/nature03443. 15829966

[6] Fong PC , Boss DS , Yap TA , Tutt A , Wu P , Mergui-Roelvink M , Mortimer P , Swaisland H , Lau A , O’Connor MJ , Ashworth A , Carmichael J , Kaye SB , et al. Inhibition of poly(ADP-ribose) polymerase in tumors from BRCA mutation carriers. N Engl J Med. 2009; 361:123–34. 10.1056/NEJMoa0900212. 19553641

[7] Satoh MS , Lindahl T . Role of poly(ADP-ribose) formation in DNA repair. Nature. 1992; 356:356–58. 10.1038/356356a0. 1549180

[8] El-Khamisy SF , Masutani M , Suzuki H , Caldecott KW . A requirement for PARP-1 for the assembly or stability of XRCC1 nuclear foci at sites of oxidative DNA damage. Nucleic Acids Res. 2003; 31:5526–33. 10.1093/nar/gkg761. 14500814 PMC206461

[9] de Murcia JM , Niedergang C , Trucco C , Ricoul M , Dutrillaux B , Mark M , Oliver FJ , Masson M , Dierich A , LeMeur M , Walztinger C , Chambon P , de Murcia G . Requirement of poly(ADP-ribose) polymerase in recovery from DNA damage in mice and in cells. Proc Natl Acad Sci U S A. 1997; 94:7303–07. 10.1073/pnas.94.14.7303. 9207086 PMC23816

[10] Wang ZQ , Stingl L , Morrison C , Jantsch M , Los M , Schulze-Osthoff K , Wagner EF . PARP is important for genomic stability but dispensable in apoptosis. Genes Dev. 1997; 11:2347–58. 10.1101/gad.11.18.2347. 9308963 PMC316515

[11] Lord CJ , Ashworth A . BRCAness revisited. Nat Rev Cancer. 2016; 16:110–20. 10.1038/nrc.2015.21. 26775620

[12] Postel-Vinay S , Bajrami I , Friboulet L , Elliott R , Fontebasso Y , Dorvault N , Olaussen KA , André F , Soria JC , Lord CJ , Ashworth A . A high-throughput screen identifies PARP1/2 inhibitors as a potential therapy for ERCC1-deficient non-small cell lung cancer. Oncogene. 2013; 32:5377–87. 10.1038/onc.2013.311. 23934192

[13] McGrail DJ , Lin CC , Garnett J , Liu Q , Mo W , Dai H , Lu Y , Yu Q , Ju Z , Yin J , Vellano CP , Hennessy B , Mills GB , Lin SY . Improved prediction of PARP inhibitor response and identification of synergizing agents through use of a novel gene expression signature generation algorithm. NPJ Syst Biol Appl. 2017; 3:8. 10.1038/s41540-017-0011-6. 28649435 PMC5445594

[14] Donawho CK , Luo Y , Luo Y , Penning TD , Bauch JL , Bouska JJ , Bontcheva-Diaz VD , Cox BF , DeWeese TL , Dillehay LE , Ferguson DC , Ghoreishi-Haack NS , Grimm DR , et al. ABT-888, an orally active poly(ADP-ribose) polymerase inhibitor that potentiates DNA-damaging agents in preclinical tumor models. Clin Cancer Res. 2007; 13:2728–37. 10.1158/1078-0432.CCR-06-3039. 17473206

[15] Michels J , Vitale I , Senovilla L , Enot DP , Garcia P , Lissa D , Olaussen KA , Brenner C , Soria JC , Castedo M , Kroemer G . Synergistic interaction between cisplatin and PARP inhibitors in non-small cell lung cancer. Cell Cycle. 2013; 12:877–83. 10.4161/cc.24034. 23428903 PMC3637345

[16] Minami D , Takigawa N , Takeda H , Takata M , Ochi N , Ichihara E , Hisamoto A , Hotta K , Tanimoto M , Kiura K . Synergistic effect of olaparib with combination of cisplatin on PTEN-deficient lung cancer cells. Mol Cancer Res. 2013; 11:140–48. 10.1158/1541-7786.MCR-12-0401. 23239809

[17] Guggenheim ER , Ondrus AE , Movassaghi M , Lippard SJ . Poly(ADP-ribose) polymerase-1 activity facilitates the dissociation of nuclear proteins from platinum-modified DNA. Bioorg Med Chem. 2008; 16:10121–28. 10.1016/j.bmc.2008.09.074. 18977144 PMC2662712

[18] Olaussen KA , Adam J , Vanhecke E , Vielh P , Pirker R , Friboulet L , Popper H , Robin A , Commo F , Thomale J , Kayitalire L , Filipits M , Le Chevalier T , et al. PARP1 impact on DNA repair of platinum adducts: preclinical and clinical read-outs. Lung Cancer. 2013; 80:216–22. 10.1016/j.lungcan.2013.01.014. 23410825

[19] Zhu G , Chang P , Lippard SJ . Recognition of platinum-DNA damage by poly(ADP-ribose) polymerase-1. Biochemistry. 2010; 49:6177–83. 10.1021/bi100775t. 20550106 PMC2912421

[20] Ramalingam SS , Blais N , Mazieres J , Reck M , Jones CM , Juhasz E , Urban L , Orlov S , Barlesi F , Kio E , Keiholz U , Qin Q , Qian J , et al. Randomized, Placebo-Controlled, Phase II Study of Veliparib in Combination with Carboplatin and Paclitaxel for Advanced/Metastatic Non-Small Cell Lung Cancer. Clin Cancer Res. 2017; 23:1937–44. 10.1158/1078-0432.CCR-15-3069. 27803064

[21] Coleman RL , Fleming GF , Brady MF , Swisher EM , Steffensen KD , Friedlander M , Okamoto A , Moore KN , Efrat Ben-Baruch N , Werner TL , Cloven NG , Oaknin A , DiSilvestro PA , et al. Veliparib with First-Line Chemotherapy and as Maintenance Therapy in Ovarian Cancer. N Engl J Med. 2019; 381:2403–15. 10.1056/NEJMoa1909707. 31562800 PMC6941439

[22] Diéras V , Han HS , Kaufman B , Wildiers H , Friedlander M , Ayoub JP , Puhalla SL , Bondarenko I , Campone M , Jakobsen EH , Jalving M , Oprean C , Palácová M , et al. Veliparib with carboplatin and paclitaxel in BRCA-mutated advanced breast cancer (BROCADE3): a randomised, double-blind, placebo-controlled, phase 3 trial. Lancet Oncol. 2020; 21:1269–82. 10.1016/S1470-2045(20)30447-2. 32861273

[23] Cheng H , Zhang Z , Borczuk A , Powell CA , Balajee AS , Lieberman HB , Halmos B . PARP inhibition selectively increases sensitivity to cisplatin in ERCC1-low non-small cell lung cancer cells. Carcinogenesis. 2013; 34:739–49. 10.1093/carcin/bgs393. 23275151 PMC3616665

[24] Miknyoczki SJ , Jones-Bolin S , Pritchard S , Hunter K , Zhao H , Wan W , Ator M , Bihovsky R , Hudkins R , Chatterjee S , Klein-Szanto A , Dionne C , Ruggeri B . Chemopotentiation of temozolomide, irinotecan, and cisplatin activity by CEP-6800, a poly(ADP-ribose) polymerase inhibitor. Mol Cancer Ther. 2003; 2:371–82. 12700281

[25] Mootha VK , Lindgren CM , Eriksson KF , Subramanian A , Sihag S , Lehar J , Puigserver P , Carlsson E , Ridderstråle M , Laurila E , Houstis N , Daly MJ , Patterson N , et al. PGC-1alpha-responsive genes involved in oxidative phosphorylation are coordinately downregulated in human diabetes. Nat Genet. 2003; 34:267–73. 10.1038/ng1180. 12808457

[26] Subramanian A , Tamayo P , Mootha VK , Mukherjee S , Ebert BL , Gillette MA , Paulovich A , Pomeroy SL , Golub TR , Lander ES , Mesirov JP . Gene set enrichment analysis: a knowledge-based approach for interpreting genome-wide expression profiles. Proc Natl Acad Sci U S A. 2005; 102:15545–50. 10.1073/pnas.0506580102. 16199517 PMC1239896

[27] Ehmsen S , Pedersen MH , Wang G , Terp MG , Arslanagic A , Hood BL , Conrads TP , Leth-Larsen R , Ditzel HJ . Increased Cholesterol Biosynthesis Is a Key Characteristic of Breast Cancer Stem Cells Influencing Patient Outcome. Cell Rep. 2019; 27:3927–38.e6. 10.1016/j.celrep.2019.05.104. 31242424

[28] Huang B , Song BL , Xu C . Cholesterol metabolism in cancer: mechanisms and therapeutic opportunities. Nat Metab. 2020; 2:132–41. 10.1038/s42255-020-0174-0. 32694690

[29] Iershov A , Nemazanyy I , Alkhoury C , Girard M , Barth E , Cagnard N , Montagner A , Chretien D , Rugarli EI , Guillou H , Pende M , Panasyuk G . The class 3 PI3K coordinates autophagy and mitochondrial lipid catabolism by controlling nuclear receptor PPARα. Nat Commun. 2019; 10:1566. 10.1038/s41467-019-09598-9. 30952952 PMC6451001

[30] Clapier CR , Cairns BR . The biology of chromatin remodeling complexes. Annu Rev Biochem. 2009; 78:273–304. 10.1146/annurev.biochem.77.062706.153223. 19355820

[31] Rother MB , van Attikum H . DNA repair goes hip-hop: SMARCA and CHD chromatin remodellers join the break dance. Philos Trans R Soc Lond B Biol Sci. 2017; 372:20160285. 10.1098/rstb.2016.0285. 28847822 PMC5577463

[32] Schiewer MJ , Knudsen KE . Transcriptional roles of PARP1 in cancer. Mol Cancer Res. 2014; 12:1069–80. 10.1158/1541-7786.MCR-13-0672. 24916104 PMC4134958

[33] Kumar M , Jaiswal RK , Prasad R , Yadav SS , Kumar A , Yadava PK , Singh RP . PARP-1 induces EMT in non-small cell lung carcinoma cells via modulating the transcription factors Smad4, p65 and ZEB1. Life Sci. 2021; 269:118994. 10.1016/j.lfs.2020.118994. 33417952

[34] Brozovic A . The relationship between platinum drug resistance and epithelial-mesenchymal transition. Arch Toxicol. 2017; 91:605–19. 10.1007/s00204-016-1912-7. 28032148

[35] Wang H , Zhang G , Zhang H , Zhang F , Zhou B , Ning F , Wang HS , Cai SH , Du J . Acquisition of epithelial-mesenchymal transition phenotype and cancer stem cell-like properties in cisplatin-resistant lung cancer cells through AKT/β-catenin/Snail signaling pathway. Eur J Pharmacol. 2014; 723:156–66. 10.1016/j.ejphar.2013.12.004. 24333218

[36] Li Y , Lv Z , He G , Wang J , Zhang X , Lu G , Ren X , Wang F , Zhu X , Ding Y , Liao W , Ding Y , Liang L . The SOX17/miR-371-5p/SOX2 axis inhibits EMT, stem cell properties and metastasis in colorectal cancer. Oncotarget. 2015; 6:9099–112. 10.18632/oncotarget.3603. 25868860 PMC4496205

[37] Lepesheva GI , Waterman MR . Sterol 14alpha-demethylase cytochrome P450 (CYP51), a P450 in all biological kingdoms. Biochim Biophys Acta. 2007; 1770:467–77. 10.1016/j.bbagen.2006.07.018. 16963187 PMC2324071

[38] Leevers SJ , Vanhaesebroeck B , Waterfield MD . Signalling through phosphoinositide 3-kinases: the lipids take centre stage. Curr Opin Cell Biol. 1999; 11:219–25. 10.1016/s0955-0674(99)80029-5. 10209156

[39] Lizardo DY , Lin YL , Gokcumen O , Atilla-Gokcumen GE . Regulation of lipids is central to replicative senescence. Mol Biosyst. 2017; 13:498–509. 10.1039/c6mb00842a. 28128379

[40] Singh P , Saxena R , Srinivas G , Pande G , Chattopadhyay A . Cholesterol biosynthesis and homeostasis in regulation of the cell cycle. PLoS One. 2013; 8:e58833. 10.1371/journal.pone.0058833. 23554937 PMC3598952

[41] Yue W , Wang T , Zachariah E , Lin Y , Yang CS , Xu Q , DiPaola RS , Tan XL . Transcriptomic analysis of pancreatic cancer cells in response to metformin and aspirin: an implication of synergy. Sci Rep. 2015; 5:13390. 10.1038/srep13390. 26294325 PMC4543968

[42] Koundouros N , Poulogiannis G . Reprogramming of fatty acid metabolism in cancer. Br J Cancer. 2020; 122:4–22. 10.1038/s41416-019-0650-z. 31819192 PMC6964678

[43] Kuzu OF , Noory MA , Robertson GP . The Role of Cholesterol in Cancer. Cancer Res. 2016; 76:2063–70. 10.1158/0008-5472.CAN-15-2613. 27197250 PMC5813477

[44] Dobin A , Davis CA , Schlesinger F , Drenkow J , Zaleski C , Jha S , Batut P , Chaisson M , Gingeras TR . STAR: ultrafast universal RNA-seq aligner. Bioinformatics. 2013; 29:15–21. 10.1093/bioinformatics/bts635. 23104886 PMC3530905

