# Supplementary material for: Transcriptomics reveals in vivo efficacy of PARP inhibitor combinatorial synergy with platinum-based chemotherapy in human non-small cell lung carcinoma models
Source: Oncotarget. 2022 Jan 3;13:1–12. doi: 10.18632/oncotarget.28162 (PMC8729805; doi:10.18632/oncotarget.28162)
Supplement: Supplementary file 1 [file oncotarget-13-28162-s001.pdf]

Transcriptomics reveals *in vivo* efficacy of PARP inhibitor combinatorial synergy with platinum-based chemotherapy in human non-small cell lung carcinoma models

SUPPLEMENTARY MATERIALS

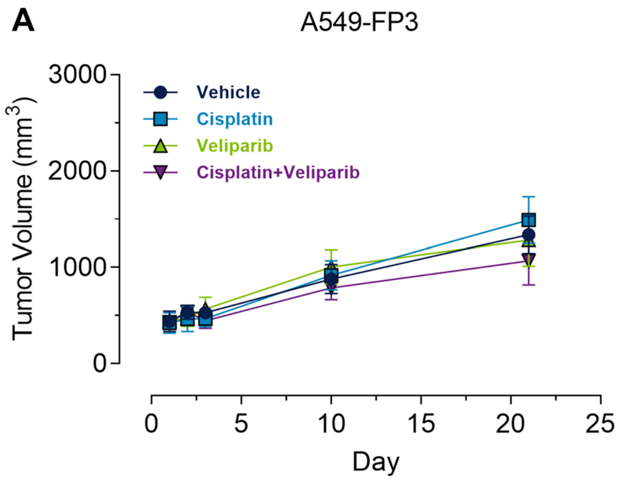

Supplementary Figure 1: *In vivo* tumor growth curves for A549-FP3 and CALU6-FP6 in mice treated with cisplatin, veliparib, and the combination. *In vivo* tumor growth curves for A549-FP3 (**A**) and CALU6-FP6 (**B**) in mice treated with vehicle, cisplatin (4.5 mg/kg IV once on day 0), veliparib (200 mg/kg/day PO QD for 21 days), and the combination. X-axis shows days since drug treatment start. Note that combination effect in Calu6-FP3 was not observed in this study. For all timepoints and treatments, n = 5 mice were used.

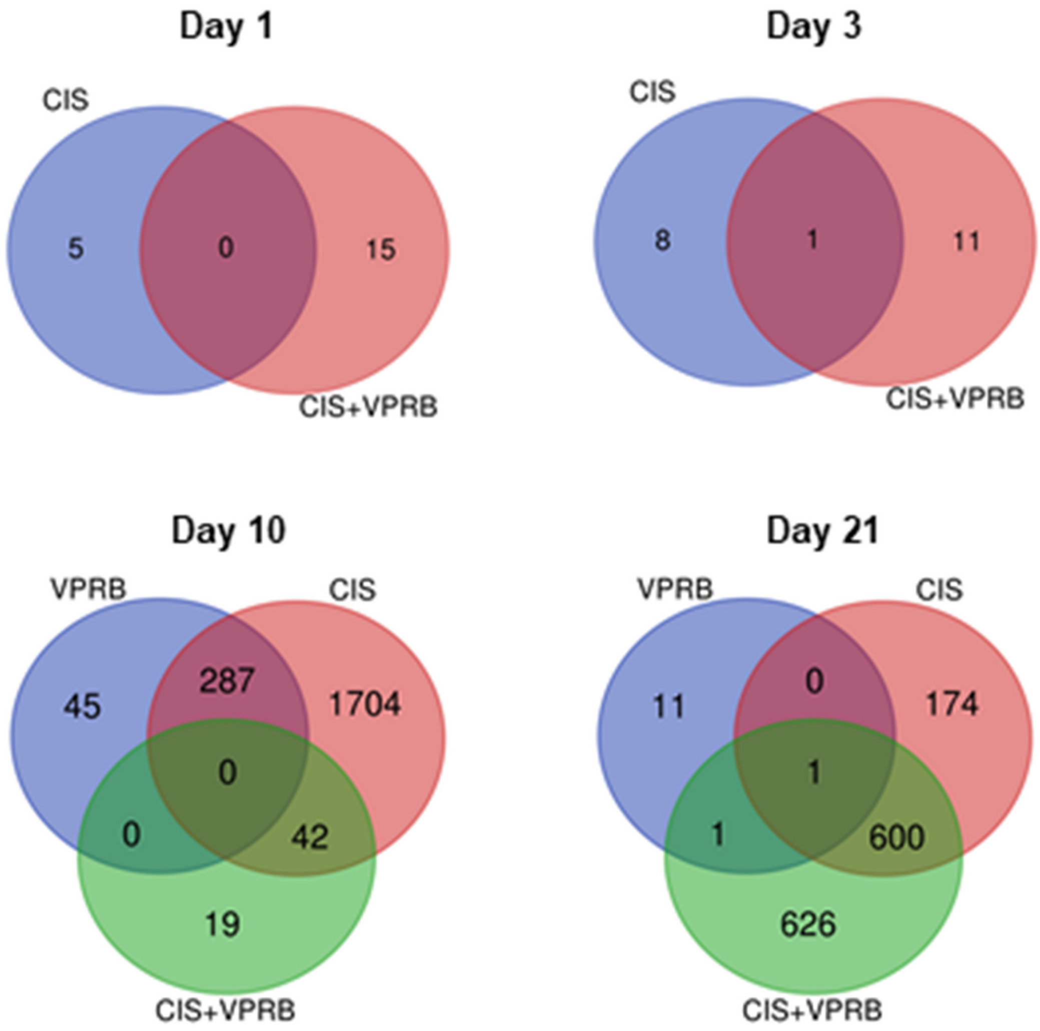

Supplementary Figure 2: Venn diagrams showing number of DEGs that are shared between cisplatin, veliparib, and cisplatin + veliparib treatment groups across time points. Significant genes were defined by differential gene expression (DGE) between no treatment and treatment arms at the indicated day with a false-discovery rate of 5% and log2 fold change values greater than 1.0/less than –1.0.

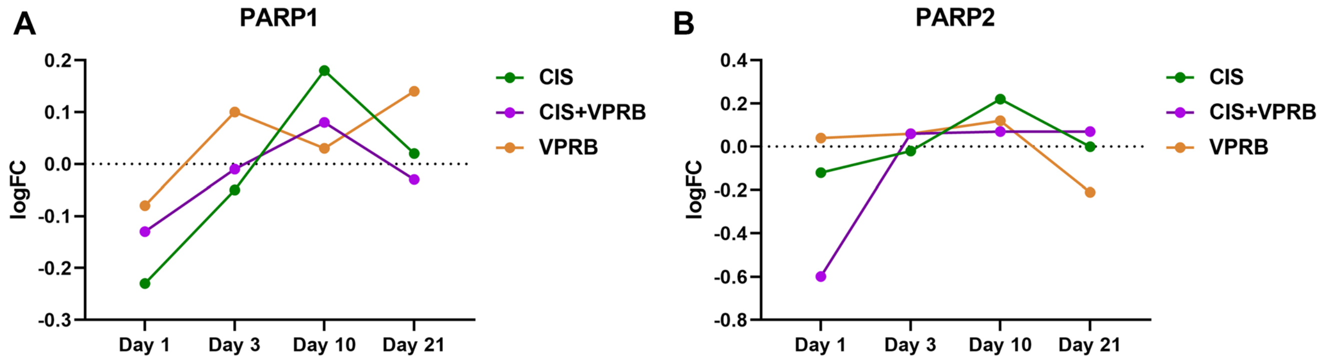

Supplementary Figure 3: Log fold change (logFC) values for differentially expressed genes in no treatment vs. cisplatin (CIS), no treatment vs. veliparib (VPRB), and no treatment vs. cisplatin + veliparib (CIS+VPRB) on days 1, 3, 10, and 21. (**A**) *PARP1*. (**B**) *PARP2*.

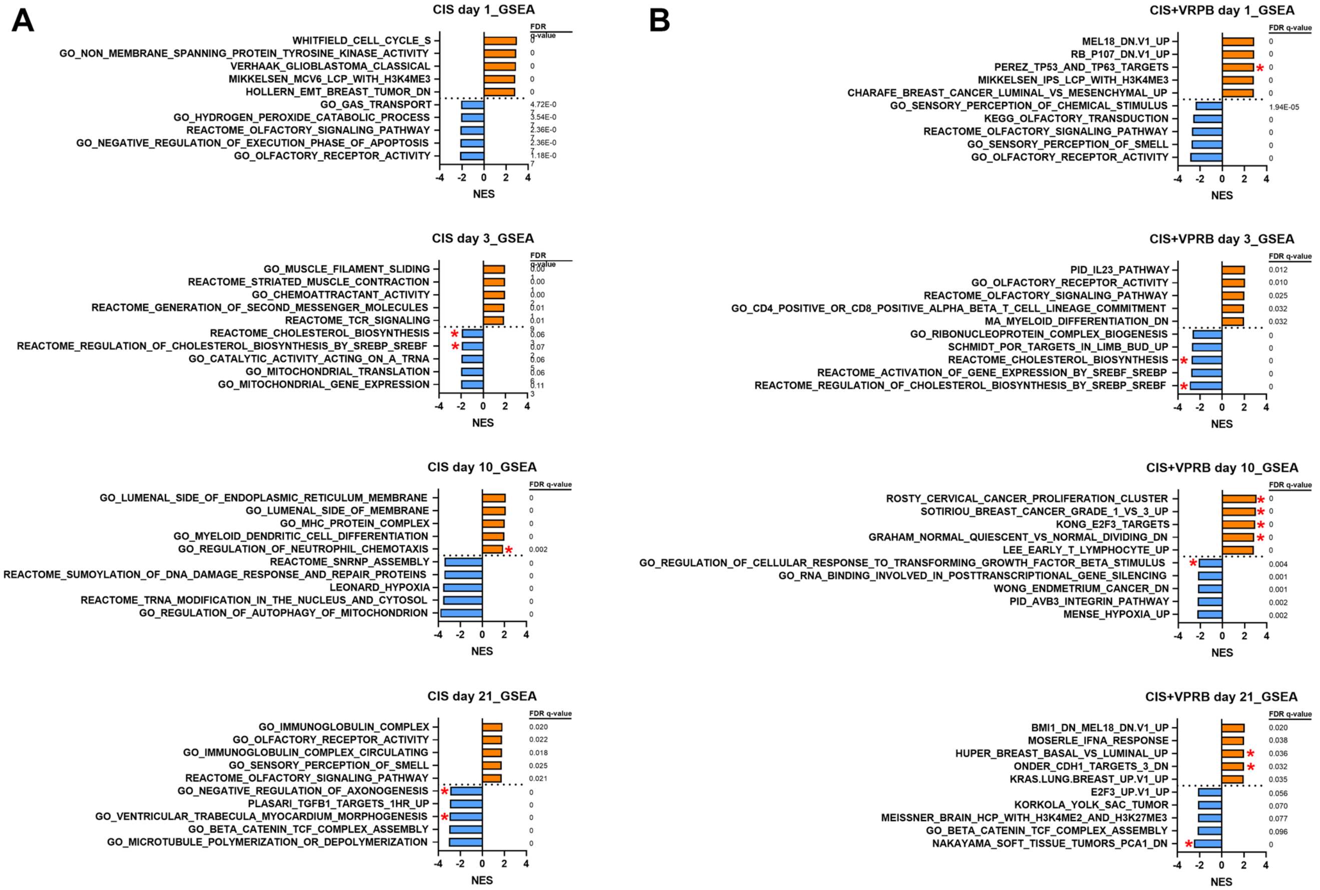

Supplementary Figure 4: GSEA gene ontology analysis showing top 5 upregulated (orange) and top 5 downregulated (blue) pathways on days 1, 3, 10, and 21. *denotes pathways shared with similar pathways from IPA at the same time point. (**A**) No treatment vs. cisplatin. (**B**) No treatment vs. cisplatin + veliparib.

Supplementary Table 1: IPA -log(*p*-values) for selected pathways involved in cell cycle and DNA repair in no treatment vs. cisplatin (CIS) and no treatment vs. cisplatin + veliparib (CIS+VPRB) on day 10

-log (*p*-values) at day 10	Role of BRCA in DNA Damage Response	Cyclins and Cell Cycle Regulation	Role of CHK Proteins in Cell Cycle Checkpoint Control	Cell Cycle: G1/S Checkpoint Regulation	Cell Cycle: G2/M DNA Damage Checkpoint Regulation	Senescence Pathway
CIS	1.18	4.76	1.37	6.72	3.03	5.92
CIS+VPRB	5.16	4.33	3.97	3.59	3.47	3.03

Supplementary Table 2: IPA -log(*p*-values) for selected pathways involved in TGF-b signaling and EMT in no treatment vs. cisplatin (CIS) and no treatment vs. cisplatin + veliparib (CIS+VPRB) on day 10

-log (*p*-values) at day 10	TGF-b Signaling	Integrin Signaling	Regulation of the Epithelial-Mesenchymal Transition Pathway	Wnt/Ca+ Pathway	Wnt/b-catenin Signaling	BMP Signaling
CIS	4.15	8.33	8.4	5.24	6.68	3.45
CIS+VPRB	3.7	0	0.888	1.11	0.78	1.15

Supplementary Table 3: IPA -log(*p*-values) for selected pathways involved in cholesterol biosynthesis in no treatment vs. cisplatin (CIS) and no treatment vs. cisplatin + veliparib (CIS+VPRB) on day 3

-log (*p*-values) at day 3	Superpathway of Cholesterol Biosynthesis	Cholesterol Biosynthesis I	Cholesterol Biosynthesis II (via 24, 25-dihydrolanosterol)	Cholesterol Biosynthesis III (via Desmosterol)	Mevalonate Pathway I	3-phosphoinositide Degradation
CIS	8.17	6.81	6.81	6.81	2.51	2.16
CIS+VPRB	8.78	7.06	7.06	7.06	2.96	2.97

Supplemntary Table 4: RNA-sequencing metrics. See Supplementary Table 4
