# Supplementary material for: Transcriptomics reveals in vivo efficacy of PARP inhibitor combinatorial synergy with platinum-based chemotherapy in human non-small cell lung carcinoma models
Source: Oncotarget. 2022 Jan 3;13:1–12. doi: 10.18632/oncotarget.28162 (PMC8729805; doi:10.18632/oncotarget.28162)
Supplement: Supplementary file 2 [file oncotarget-13-28162-s002.docx]

**Supplementary Table 4: RNA-sequencing metrics**

| **Cell line** | **Treatment Arm** | **Xenograft collection timepoint (Day)** | **Avg. GC** | **% Duplicates** | **Insert Size** | **% Coding** | **% UTR** | **% Intronic** | **% Intergenic** | **Total seqs (Million)** | **% Reads Aligned** | **Reads Aligned (Million)** |
| --- | --- | --- | --- | --- | --- | --- | --- | --- | --- | --- | --- | --- |
| A549-FP3 | CISPLATIN | 1 | 0.56 | 0.417 | 151 | 0.306 | 0.339 | 0.237 | 0.117 | 57.6 | 0.579 | 23.6 |
| A549-FP3 | CISPLATIN | 1 | 0.53 | 0.442 | 166 | 0.316 | 0.31 | 0.263 | 0.112 | 119.1 | 0.801 | 50.4 |
| A549-FP3 | CISPLATIN | 1 | 0.54 | 0.429 | 157 | 0.354 | 0.361 | 0.191 | 0.094 | 89.5 | 0.779 | 37 |
| A549-FP3 | CISPLATIN | 1 | 0.58 | 0.468 | 151 | 0.322 | 0.38 | 0.156 | 0.142 | 45.4 | 0.59 | 17.3 |
| A549-FP3 | CISPLATIN_VELIPARIB | 1 | 0.52 | 0.38 | 159 | 0.358 | 0.326 | 0.229 | 0.086 | 93 | 0.821 | 40.4 |
| A549-FP3 | CISPLATIN_VELIPARIB | 1 | 0.55 | 0.482 | 158 | 0.302 | 0.312 | 0.206 | 0.18 | 100.9 | 0.668 | 38.2 |
| A549-FP3 | CISPLATIN_VELIPARIB | 1 | 0.56 | 0.421 | 163 | 0.356 | 0.338 | 0.188 | 0.118 | 87.4 | 0.697 | 36.4 |
| A549-FP3 | CISPLATIN_VELIPARIB | 1 | 0.54 | 0.415 | 164 | 0.365 | 0.333 | 0.187 | 0.115 | 61.1 | 0.733 | 25.4 |
| A549-FP3 | CISPLATIN_VELIPARIB | 1 | 0.55 | 0.438 | 160 | 0.344 | 0.355 | 0.192 | 0.109 | 83.3 | 0.768 | 34.3 |
| A549-FP3 | NO TREATMENT | 1 | 0.55 | 0.538 | 138 | 0.265 | 0.321 | 0.208 | 0.207 | 139.3 | 0.597 | 46.3 |
| A549-FP3 | NO TREATMENT | 1 | 0.57 | 0.583 | 128 | 0.251 | 0.326 | 0.174 | 0.249 | 122.8 | 0.508 | 36.2 |
| A549-FP3 | NO TREATMENT | 1 | 0.57 | 0.687 | 129 | 0.176 | 0.335 | 0.16 | 0.328 | 133.1 | 0.434 | 31.4 |
| A549-FP3 | NO TREATMENT | 1 | 0.55 | 0.548 | 143 | 0.309 | 0.331 | 0.177 | 0.182 | 129.7 | 0.641 | 44.9 |
| A549-FP3 | VELIPARIB | 1 | 0.56 | 0.663 | 129 | 0.196 | 0.291 | 0.148 | 0.366 | 167.6 | 0.457 | 41.6 |
| A549-FP3 | VELIPARIB | 1 | 0.57 | 0.723 | 132 | 0.147 | 0.302 | 0.141 | 0.41 | 158.6 | 0.405 | 34.8 |
| A549-FP3 | VELIPARIB | 1 | 0.56 | 0.581 | 146 | 0.228 | 0.328 | 0.195 | 0.249 | 119.4 | 0.497 | 35.6 |
| A549-FP3 | CISPLATIN | 2 | 0.56 | 0.397 | 159 | 0.361 | 0.362 | 0.173 | 0.104 | 45.2 | 0.705 | 18.6 |
| A549-FP3 | CISPLATIN | 2 | 0.54 | 0.424 | 162 | 0.339 | 0.345 | 0.186 | 0.131 | 65.2 | 0.748 | 26.3 |
| A549-FP3 | CISPLATIN | 2 | 0.53 | 0.47 | 166 | 0.339 | 0.359 | 0.193 | 0.11 | 93.1 | 0.766 | 37.9 |
| A549-FP3 | CISPLATIN | 2 | 0.56 | 0.597 | 150 | 0.231 | 0.289 | 0.13 | 0.351 | 88.3 | 0.499 | 24.4 |
| A549-FP3 | CISPLATIN | 2 | 0.56 | 0.427 | 147 | 0.348 | 0.373 | 0.171 | 0.108 | 50.7 | 0.603 | 20.4 |
| A549-FP3 | CISPLATIN_VELIPARIB | 2 | 0.53 | 0.42 | 173 | 0.397 | 0.364 | 0.17 | 0.07 | 71.9 | 0.726 | 31.1 |
| A549-FP3 | CISPLATIN_VELIPARIB | 2 | 0.56 | 0.42 | 157 | 0.352 | 0.365 | 0.18 | 0.103 | 60.8 | 0.589 | 25.1 |
| A549-FP3 | CISPLATIN_VELIPARIB | 2 | 0.54 | 0.44 | 164 | 0.363 | 0.355 | 0.189 | 0.093 | 103.5 | 0.777 | 43.4 |
| A549-FP3 | CISPLATIN_VELIPARIB | 2 | 0.53 | 0.468 | 168 | 0.348 | 0.355 | 0.198 | 0.099 | 118.8 | 0.749 | 49.2 |
| A549-FP3 | CISPLATIN_VELIPARIB | 2 | 0.56 | 0.488 | 159 | 0.336 | 0.387 | 0.178 | 0.1 | 101.2 | 0.735 | 40.3 |
| A549-FP3 | NO TREATMENT | 2 | 0.53 | 0.402 | 157 | 0.352 | 0.333 | 0.213 | 0.102 | 89.4 | 0.777 | 37.7 |
| A549-FP3 | NO TREATMENT | 2 | 0.56 | 0.424 | 159 | 0.351 | 0.366 | 0.168 | 0.115 | 54.3 | 0.665 | 22 |
| A549-FP3 | NO TREATMENT | 2 | 0.56 | 0.497 | 162 | 0.34 | 0.367 | 0.191 | 0.103 | 102.6 | 0.725 | 41.8 |
| A549-FP3 | NO TREATMENT | 2 | 0.54 | 0.476 | 157 | 0.401 | 0.351 | 0.168 | 0.08 | 142.9 | 0.799 | 60.2 |
| A549-FP3 | NO TREATMENT | 2 | 0.54 | 0.419 | 162 | 0.383 | 0.356 | 0.174 | 0.087 | 78.7 | 0.802 | 33.1 |
| A549-FP3 | VELIPARIB | 2 | 0.56 | 0.447 | 165 | 0.321 | 0.378 | 0.195 | 0.106 | 55.5 | 0.74 | 22.7 |
| A549-FP3 | VELIPARIB | 2 | 0.54 | 0.429 | 170 | 0.328 | 0.374 | 0.208 | 0.09 | 62.7 | 0.746 | 26.2 |
| A549-FP3 | VELIPARIB | 2 | 0.53 | 0.392 | 171 | 0.36 | 0.337 | 0.213 | 0.09 | 100.1 | 0.806 | 43 |
| A549-FP3 | VELIPARIB | 2 | 0.55 | 0.444 | 156 | 0.356 | 0.364 | 0.174 | 0.106 | 65.8 | 0.662 | 27 |
| A549-FP3 | VELIPARIB | 2 | 0.57 | 0.638 | 149 | 0.2 | 0.281 | 0.139 | 0.38 | 76.8 | 0.421 | 19.6 |
| A549-FP3 | CISPLATIN | 3 | 0.56 | 0.43 | 151 | 0.291 | 0.339 | 0.226 | 0.144 | 50.8 | 0.685 | 20.1 |
| A549-FP3 | CISPLATIN | 3 | 0.56 | 0.439 | 175 | 0.327 | 0.355 | 0.199 | 0.119 | 70.4 | 0.697 | 29 |
| A549-FP3 | CISPLATIN | 3 | 0.54 | 0.406 | 178 | 0.374 | 0.344 | 0.191 | 0.091 | 78.4 | 0.796 | 33.5 |
| A549-FP3 | CISPLATIN | 3 | 0.56 | 0.429 | 169 | 0.347 | 0.345 | 0.184 | 0.124 | 69.2 | 0.631 | 28.4 |
| A549-FP3 | CISPLATIN | 3 | 0.56 | 0.448 | 157 | 0.302 | 0.342 | 0.208 | 0.149 | 77.4 | 0.667 | 30.5 |
| A549-FP3 | CISPLATIN_VELIPARIB | 3 | 0.56 | 0.426 | 152 | 0.337 | 0.343 | 0.203 | 0.118 | 70.4 | 0.633 | 28.9 |
| A549-FP3 | CISPLATIN_VELIPARIB | 3 | 0.54 | 0.4 | 151 | 0.311 | 0.308 | 0.257 | 0.124 | 105 | 0.791 | 43.8 |
| A549-FP3 | CISPLATIN_VELIPARIB | 3 | 0.54 | 0.405 | 162 | 0.33 | 0.322 | 0.239 | 0.109 | 94.9 | 0.786 | 39.8 |
| A549-FP3 | CISPLATIN_VELIPARIB | 3 | 0.56 | 0.454 | 157 | 0.263 | 0.312 | 0.254 | 0.171 | 81.8 | 0.72 | 31.9 |
| A549-FP3 | CISPLATIN_VELIPARIB | 3 | 0.55 | 0.551 | 171 | 0.371 | 0.32 | 0.214 | 0.095 | 87.9 | 0.8 | 37.7 |
| A549-FP3 | NO TREATMENT | 3 | 0.56 | 0.43 | 152 | 0.324 | 0.347 | 0.204 | 0.126 | 57.5 | 0.608 | 23.3 |
| A549-FP3 | NO TREATMENT | 3 | 0.54 | 0.43 | 167 | 0.368 | 0.337 | 0.2 | 0.095 | 97.7 | 0.796 | 41.2 |
| A549-FP3 | NO TREATMENT | 3 | 0.56 | 0.451 | 159 | 0.319 | 0.349 | 0.208 | 0.124 | 78.5 | 0.733 | 31.9 |
| A549-FP3 | NO TREATMENT | 3 | 0.55 | 0.444 | 159 | 0.381 | 0.351 | 0.176 | 0.092 | 92.2 | 0.734 | 38.6 |
| A549-FP3 | NO TREATMENT | 3 | 0.56 | 0.457 | 163 | 0.31 | 0.333 | 0.214 | 0.143 | 90.2 | 0.7 | 36.3 |
| A549-FP3 | VELIPARIB | 3 | 0.55 | 0.43 | 163 | 0.39 | 0.346 | 0.178 | 0.086 | 98.9 | 0.752 | 41.8 |
| A549-FP3 | VELIPARIB | 3 | 0.55 | 0.375 | 155 | 0.34 | 0.328 | 0.216 | 0.115 | 45.1 | 0.663 | 18.7 |
| A549-FP3 | VELIPARIB | 3 | 0.53 | 0.401 | 164 | 0.34 | 0.324 | 0.237 | 0.1 | 87 | 0.799 | 37.2 |
| A549-FP3 | VELIPARIB | 3 | 0.54 | 0.425 | 165 | 0.351 | 0.331 | 0.222 | 0.096 | 97.8 | 0.779 | 41.6 |
| A549-FP3 | VELIPARIB | 3 | 0.54 | 0.458 | 163 | 0.389 | 0.326 | 0.196 | 0.088 | 51.2 | 0.709 | 22.2 |
| A549-FP3 | CISPLATIN | 10 | 0.55 | 0.486 | 170 | 0.395 | 0.34 | 0.177 | 0.087 | 126.4 | 0.793 | 54 |
| A549-FP3 | CISPLATIN | 10 | 0.56 | 0.43 | 163 | 0.35 | 0.346 | 0.184 | 0.12 | 70.9 | 0.734 | 29.1 |
| A549-FP3 | CISPLATIN | 10 | 0.55 | 0.478 | 152 | 0.347 | 0.357 | 0.186 | 0.11 | 103 | 0.702 | 41.5 |
| A549-FP3 | CISPLATIN | 10 | 0.55 | 0.472 | 167 | 0.414 | 0.376 | 0.138 | 0.072 | 75.9 | 0.7 | 32.2 |
| A549-FP3 | CISPLATIN | 10 | 0.58 | 0.474 | 152 | 0.341 | 0.366 | 0.159 | 0.135 | 72.5 | 0.569 | 28.4 |
| A549-FP3 | CISPLATIN_VELIPARIB | 10 | 0.55 | 0.495 | 163 | 0.387 | 0.366 | 0.161 | 0.087 | 91.7 | 0.79 | 38.4 |
| A549-FP3 | CISPLATIN_VELIPARIB | 10 | 0.54 | 0.42 | 165 | 0.402 | 0.358 | 0.162 | 0.078 | 80.1 | 0.781 | 34.1 |
| A549-FP3 | CISPLATIN_VELIPARIB | 10 | 0.55 | 0.496 | 170 | 0.45 | 0.361 | 0.129 | 0.061 | 156.8 | 0.821 | 68.1 |
| A549-FP3 | CISPLATIN_VELIPARIB | 10 | 0.56 | 0.443 | 168 | 0.402 | 0.378 | 0.137 | 0.083 | 61.3 | 0.766 | 26 |
| A549-FP3 | CISPLATIN_VELIPARIB | 10 | 0.55 | 0.443 | 158 | 0.374 | 0.355 | 0.181 | 0.09 | 78.3 | 0.702 | 32.8 |
| A549-FP3 | NO TREATMENT | 10 | 0.57 | 0.445 | 155 | 0.394 | 0.355 | 0.155 | 0.096 | 54.5 | 0.604 | 22.4 |
| A549-FP3 | NO TREATMENT | 10 | 0.57 | 0.416 | 153 | 0.381 | 0.364 | 0.147 | 0.107 | 48.8 | 0.594 | 20 |
| A549-FP3 | NO TREATMENT | 10 | 0.53 | 0.404 | 165 | 0.409 | 0.35 | 0.171 | 0.07 | 77.2 | 0.782 | 33.3 |
| A549-FP3 | NO TREATMENT | 10 | 0.56 | 0.432 | 155 | 0.368 | 0.352 | 0.177 | 0.103 | 55.3 | 0.57 | 23.1 |
| A549-FP3 | NO TREATMENT | 10 | 0.54 | 0.431 | 160 | 0.421 | 0.347 | 0.155 | 0.077 | 103.8 | 0.817 | 44.6 |
| A549-FP3 | VELIPARIB | 10 | 0.55 | 0.435 | 166 | 0.381 | 0.366 | 0.157 | 0.096 | 82.3 | 0.768 | 34.5 |
| A549-FP3 | VELIPARIB | 10 | 0.56 | 0.488 | 165 | 0.37 | 0.356 | 0.169 | 0.104 | 82 | 0.71 | 33.6 |
| A549-FP3 | VELIPARIB | 10 | 0.54 | 0.426 | 160 | 0.404 | 0.348 | 0.163 | 0.085 | 79.7 | 0.741 | 33.7 |
| A549-FP3 | VELIPARIB | 10 | 0.56 | 0.507 | 162 | 0.397 | 0.37 | 0.149 | 0.084 | 85.5 | 0.801 | 35.9 |
| A549-FP3 | VELIPARIB | 10 | 0.56 | 0.494 | 166 | 0.381 | 0.368 | 0.152 | 0.098 | 85 | 0.708 | 34.7 |
| A549-FP3 | CISPLATIN | 21 | 0.55 | 0.432 | 168 | 0.384 | 0.367 | 0.166 | 0.083 | 97 | 0.793 | 40.7 |
| A549-FP3 | CISPLATIN | 21 | 0.55 | 0.388 | 159 | 0.343 | 0.351 | 0.198 | 0.108 | 44.5 | 0.678 | 18.4 |
| A549-FP3 | CISPLATIN | 21 | 0.57 | 0.423 | 156 | 0.315 | 0.339 | 0.211 | 0.135 | 54.4 | 0.63 | 21.7 |
| A549-FP3 | CISPLATIN | 21 | 0.56 | 0.488 | 167 | 0.333 | 0.333 | 0.221 | 0.112 | 97.2 | 0.71 | 39.8 |
| A549-FP3 | CISPLATIN | 21 | 0.56 | 0.458 | 162 | 0.337 | 0.355 | 0.192 | 0.116 | 90.6 | 0.69 | 36.9 |
| A549-FP3 | CISPLATIN | 21 | 0.57 | 0.467 | 142 | 0.3 | 0.356 | 0.209 | 0.136 | 78.9 | 0.627 | 31 |
| A549-FP3 | CISPLATIN_VELIPARIB | 21 | 0.56 | 0.425 | 162 | 0.367 | 0.38 | 0.157 | 0.097 | 66.1 | 0.676 | 27.2 |
| A549-FP3 | CISPLATIN_VELIPARIB | 21 | 0.53 | 0.419 | 179 | 0.366 | 0.351 | 0.203 | 0.08 | 74.8 | 0.732 | 32.4 |
| A549-FP3 | CISPLATIN_VELIPARIB | 21 | 0.55 | 0.454 | 155 | 0.343 | 0.348 | 0.207 | 0.101 | 95.3 | 0.753 | 39 |
| A549-FP3 | CISPLATIN_VELIPARIB | 21 | 0.57 | 0.439 | 154 | 0.321 | 0.368 | 0.187 | 0.123 | 52.4 | 0.703 | 20.5 |
| A549-FP3 | CISPLATIN_VELIPARIB | 21 | 0.56 | 0.467 | 154 | 0.323 | 0.394 | 0.171 | 0.112 | 75.9 | 0.693 | 29.9 |
| A549-FP3 | NO TREATMENT | 21 | 0.56 | 0.43 | 168 | 0.374 | 0.33 | 0.194 | 0.102 | 50.7 | 0.668 | 21.3 |
| A549-FP3 | NO TREATMENT | 21 | 0.56 | 0.539 | 182 | 0.314 | 0.337 | 0.222 | 0.126 | 72.3 | 0.709 | 29.9 |
| A549-FP3 | NO TREATMENT | 21 | 0.56 | 0.416 | 157 | 0.279 | 0.325 | 0.256 | 0.14 | 59.8 | 0.609 | 24 |
| A549-FP3 | NO TREATMENT | 21 | 0.59 | 0.614 | 144 | 0.32 | 0.36 | 0.174 | 0.145 | 77.5 | 0.701 | 29.1 |
| A549-FP3 | NO TREATMENT | 21 | 0.56 | 0.414 | 166 | 0.348 | 0.34 | 0.201 | 0.112 | 65.2 | 0.761 | 27.3 |
| A549-FP3 | VELIPARIB | 21 | 0.55 | 0.467 | 168 | 0.336 | 0.336 | 0.23 | 0.098 | 99.6 | 0.783 | 42.1 |
| A549-FP3 | VELIPARIB | 21 | 0.57 | 0.47 | 155 | 0.302 | 0.32 | 0.234 | 0.145 | 71.2 | 0.69 | 28.5 |
| A549-FP3 | VELIPARIB | 21 | 0.57 | 0.429 | 155 | 0.328 | 0.338 | 0.211 | 0.124 | 64.1 | 0.623 | 26.2 |
| A549-FP3 | VELIPARIB | 21 | 0.55 | 0.37 | 163 | 0.322 | 0.329 | 0.239 | 0.11 | 44.5 | 0.676 | 18.8 |
| A549-FP3 | VELIPARIB | 21 | 0.54 | 0.426 | 169 | 0.369 | 0.344 | 0.21 | 0.077 | 88.4 | 0.803 | 38.3 |
| CALU6-FP6 | CISPLATIN | 1 | 0.51 | 0.378 | 176 | 0.467 | 0.329 | 0.139 | 0.065 | 107.9 | 0.828 | 49 |
| CALU6-FP6 | CISPLATIN | 1 | 0.51 | 0.339 | 179 | 0.508 | 0.286 | 0.145 | 0.061 | 98.2 | 0.792 | 45.8 |
| CALU6-FP6 | CISPLATIN | 1 | 0.5 | 0.333 | 175 | 0.46 | 0.292 | 0.177 | 0.071 | 113.2 | 0.84 | 52.2 |
| CALU6-FP6 | CISPLATIN | 1 | 0.52 | 0.354 | 175 | 0.488 | 0.32 | 0.123 | 0.068 | 78.2 | 0.759 | 35.2 |
| CALU6-FP6 | CISPLATIN | 1 | 0.5 | 0.372 | 177 | 0.454 | 0.319 | 0.165 | 0.063 | 74.7 | 0.847 | 34.3 |
| CALU6-FP6 | CISPLATIN_VELIPARIB | 1 | 0.52 | 0.392 | 180 | 0.493 | 0.322 | 0.123 | 0.062 | 110.1 | 0.834 | 50.1 |
| CALU6-FP6 | CISPLATIN_VELIPARIB | 1 | 0.52 | 0.353 | 188 | 0.468 | 0.335 | 0.136 | 0.06 | 84.2 | 0.822 | 38.2 |
| CALU6-FP6 | CISPLATIN_VELIPARIB | 1 | 0.51 | 0.359 | 206 | 0.533 | 0.285 | 0.127 | 0.055 | 94 | 0.837 | 44.2 |
| CALU6-FP6 | CISPLATIN_VELIPARIB | 1 | 0.52 | 0.363 | 172 | 0.469 | 0.336 | 0.135 | 0.061 | 79.9 | 0.816 | 36.1 |
| CALU6-FP6 | CISPLATIN_VELIPARIB | 1 | 0.54 | 0.344 | 166 | 0.448 | 0.335 | 0.138 | 0.079 | 34.2 | 0.595 | 15.1 |
| CALU6-FP6 | NO TREATMENT | 1 | 0.58 | 0.719 | 138 | 0.281 | 0.328 | 0.1 | 0.291 | 67.9 | 0.367 | 17.4 |
| CALU6-FP6 | NO TREATMENT | 1 | 0.53 | 0.455 | 225 | 0.548 | 0.298 | 0.097 | 0.056 | 50.9 | 0.619 | 23.5 |
| CALU6-FP6 | NO TREATMENT | 1 | 0.54 | 0.557 | 146 | 0.342 | 0.322 | 0.123 | 0.213 | 111.9 | 0.609 | 36.9 |
| CALU6-FP6 | NO TREATMENT | 1 | 0.55 | 0.621 | 139 | 0.28 | 0.342 | 0.128 | 0.25 | 126.2 | 0.539 | 36.2 |
| CALU6-FP6 | NO TREATMENT | 1 | 0.52 | 0.385 | 210 | 0.531 | 0.307 | 0.108 | 0.054 | 58.9 | 0.668 | 27.2 |
| CALU6-FP6 | VELIPARIB | 1 | 0.53 | 0.413 | 208 | 0.54 | 0.305 | 0.099 | 0.055 | 59.1 | 0.669 | 27.1 |
| CALU6-FP6 | VELIPARIB | 1 | 0.55 | 0.586 | 140 | 0.308 | 0.331 | 0.125 | 0.235 | 124.9 | 0.57 | 38 |
| CALU6-FP6 | VELIPARIB | 1 | 0.54 | 0.545 | 139 | 0.329 | 0.318 | 0.135 | 0.218 | 111.3 | 0.57 | 36.4 |
| CALU6-FP6 | VELIPARIB | 1 | 0.56 | 0.676 | 139 | 0.245 | 0.359 | 0.104 | 0.292 | 126.5 | 0.459 | 31.3 |
| CALU6-FP6 | VELIPARIB | 1 | 0.56 | 0.616 | 142 | 0.261 | 0.33 | 0.135 | 0.274 | 160.5 | 0.504 | 44.1 |
| CALU6-FP6 | CISPLATIN_VELIPARIB | 2 | 0.51 | 0.393 | 188 | 0.483 | 0.295 | 0.156 | 0.066 | 73.9 | 0.834 | 34.3 |
| CALU6-FP6 | CISPLATIN_VELIPARIB | 2 | 0.55 | 0.409 | 166 | 0.427 | 0.319 | 0.153 | 0.101 | 64 | 0.616 | 28 |
| CALU6-FP6 | CISPLATIN_VELIPARIB | 2 | 0.53 | 0.452 | 197 | 0.532 | 0.291 | 0.114 | 0.063 | 61 | 0.72 | 28.1 |
| CALU6-FP6 | CISPLATIN_VELIPARIB | 2 | 0.54 | 0.383 | 171 | 0.437 | 0.314 | 0.161 | 0.088 | 67.4 | 0.719 | 30 |
| CALU6-FP6 | CISPLATIN_VELIPARIB | 2 | 0.54 | 0.476 | 172 | 0.508 | 0.297 | 0.129 | 0.066 | 81.4 | 0.835 | 37.2 |
| CALU6-FP6 | VELIPARIB | 2 | 0.55 | 0.35 | 151 | 0.455 | 0.333 | 0.126 | 0.086 | 37.8 | 0.577 | 16.6 |
| CALU6-FP6 | VELIPARIB | 2 | 0.53 | 0.421 | 173 | 0.438 | 0.326 | 0.154 | 0.082 | 107.2 | 0.707 | 47.8 |
| CALU6-FP6 | VELIPARIB | 2 | 0.53 | 0.369 | 182 | 0.48 | 0.33 | 0.121 | 0.069 | 76.6 | 0.78 | 34.7 |
| CALU6-FP6 | VELIPARIB | 2 | 0.52 | 0.363 | 168 | 0.475 | 0.318 | 0.145 | 0.063 | 89.6 | 0.846 | 40.7 |
| CALU6-FP6 | VELIPARIB | 2 | 0.55 | 0.384 | 176 | 0.474 | 0.305 | 0.123 | 0.098 | 64.4 | 0.632 | 28.7 |
| CALU6-FP6 | CISPLATIN | 3 | 0.54 | 0.494 | 173 | 0.503 | 0.326 | 0.115 | 0.056 | 100.8 | 0.813 | 45.7 |
| CALU6-FP6 | CISPLATIN | 3 | 0.53 | 0.384 | 162 | 0.424 | 0.337 | 0.146 | 0.093 | 66.2 | 0.709 | 28.7 |
| CALU6-FP6 | CISPLATIN | 3 | 0.53 | 0.36 | 168 | 0.431 | 0.334 | 0.159 | 0.076 | 68.7 | 0.73 | 30.7 |
| CALU6-FP6 | CISPLATIN | 3 | 0.54 | 0.442 | 177 | 0.438 | 0.333 | 0.134 | 0.096 | 76.1 | 0.602 | 32.4 |
| CALU6-FP6 | CISPLATIN | 3 | 0.53 | 0.452 | 180 | 0.46 | 0.332 | 0.14 | 0.069 | 97.8 | 0.68 | 43.6 |
| CALU6-FP6 | CISPLATIN_VELIPARIB | 3 | 0.54 | 0.492 | 166 | 0.446 | 0.32 | 0.155 | 0.079 | 90.9 | 0.83 | 40.2 |
| CALU6-FP6 | CISPLATIN_VELIPARIB | 3 | 0.54 | 0.395 | 179 | 0.456 | 0.302 | 0.158 | 0.084 | 50.6 | 0.678 | 22.6 |
| CALU6-FP6 | CISPLATIN_VELIPARIB | 3 | 0.53 | 0.362 | 191 | 0.473 | 0.353 | 0.112 | 0.062 | 36.2 | 0.682 | 16.3 |
| CALU6-FP6 | CISPLATIN_VELIPARIB | 3 | 0.52 | 0.386 | 167 | 0.455 | 0.346 | 0.124 | 0.075 | 86.3 | 0.776 | 37.8 |
| CALU6-FP6 | CISPLATIN_VELIPARIB | 3 | 0.53 | 0.526 | 183 | 0.481 | 0.337 | 0.122 | 0.06 | 74.7 | 0.808 | 33.8 |
| CALU6-FP6 | NO TREATMENT | 3 | 0.52 | 0.402 | 192 | 0.477 | 0.341 | 0.122 | 0.06 | 101 | 0.828 | 45.6 |
| CALU6-FP6 | NO TREATMENT | 3 | 0.54 | 0.354 | 157 | 0.444 | 0.329 | 0.149 | 0.078 | 56 | 0.675 | 25 |
| CALU6-FP6 | NO TREATMENT | 3 | 0.52 | 0.479 | 192 | 0.471 | 0.315 | 0.151 | 0.064 | 88.2 | 0.808 | 40.3 |
| CALU6-FP6 | NO TREATMENT | 3 | 0.53 | 0.357 | 167 | 0.433 | 0.329 | 0.151 | 0.086 | 69.8 | 0.742 | 30.7 |
| CALU6-FP6 | NO TREATMENT | 3 | 0.53 | 0.381 | 177 | 0.49 | 0.346 | 0.107 | 0.058 | 89.3 | 0.797 | 40 |
| CALU6-FP6 | VELIPARIB | 3 | 0.52 | 0.36 | 184 | 0.481 | 0.341 | 0.118 | 0.059 | 81.9 | 0.768 | 36.9 |
| CALU6-FP6 | VELIPARIB | 3 | 0.51 | 0.388 | 181 | 0.455 | 0.317 | 0.163 | 0.065 | 103.1 | 0.803 | 47.1 |
| CALU6-FP6 | VELIPARIB | 3 | 0.53 | 0.405 | 180 | 0.471 | 0.349 | 0.112 | 0.068 | 86.9 | 0.808 | 38.8 |
| CALU6-FP6 | VELIPARIB | 3 | 0.52 | 0.362 | 180 | 0.486 | 0.337 | 0.119 | 0.057 | 53.3 | 0.718 | 24.2 |
| CALU6-FP6 | VELIPARIB | 3 | 0.53 | 0.391 | 174 | 0.508 | 0.339 | 0.098 | 0.055 | 67.5 | 0.72 | 30.4 |
| CALU6-FP6 | CISPLATIN | 10 | 0.55 | 0.45 | 185 | 0.531 | 0.282 | 0.087 | 0.1 | 33.4 | 0.301 | 14.3 |
| CALU6-FP6 | CISPLATIN | 10 | 0.6 | 0.644 | 153 | 0.331 | 0.4 | 0.102 | 0.168 | 41.8 | 0.285 | 14.1 |
| CALU6-FP6 | CISPLATIN | 10 | 0.57 | 0.494 | 158 | 0.425 | 0.362 | 0.115 | 0.099 | 42.2 | 0.377 | 16.4 |
| CALU6-FP6 | CISPLATIN | 10 | 0.57 | 0.441 | 154 | 0.457 | 0.311 | 0.101 | 0.131 | 41.8 | 0.394 | 17 |
| CALU6-FP6 | CISPLATIN_VELIPARIB | 10 | 0.55 | 0.572 | 173 | 0.518 | 0.264 | 0.13 | 0.088 | 68.5 | 0.692 | 30.7 |
| CALU6-FP6 | CISPLATIN_VELIPARIB | 10 | 0.51 | 0.373 | 191 | 0.476 | 0.298 | 0.159 | 0.067 | 79.9 | 0.747 | 37 |
| CALU6-FP6 | CISPLATIN_VELIPARIB | 10 | 0.53 | 0.329 | 190 | 0.511 | 0.297 | 0.118 | 0.075 | 45.8 | 0.614 | 21 |
| CALU6-FP6 | NO TREATMENT | 10 | 0.58 | 0.424 | 163 | 0.408 | 0.31 | 0.119 | 0.163 | 27.9 | 0.432 | 11.1 |
| CALU6-FP6 | NO TREATMENT | 10 | 0.55 | 0.47 | 161 | 0.509 | 0.295 | 0.113 | 0.082 | 102.7 | 0.695 | 45.1 |
| CALU6-FP6 | NO TREATMENT | 10 | 0.53 | 0.447 | 177 | 0.452 | 0.312 | 0.155 | 0.081 | 56.3 | 0.735 | 25.4 |
| CALU6-FP6 | NO TREATMENT | 10 | 0.59 | 0.478 | 149 | 0.371 | 0.324 | 0.124 | 0.181 | 53.8 | 0.518 | 20.1 |
| CALU6-FP6 | NO TREATMENT | 10 | 0.57 | 0.503 | 160 | 0.455 | 0.312 | 0.109 | 0.123 | 68.3 | 0.596 | 28 |
| CALU6-FP6 | VELIPARIB | 10 | 0.61 | 0.643 | 138 | 0.249 | 0.263 | 0.11 | 0.379 | 54.5 | 0.363 | 14.2 |
| CALU6-FP6 | VELIPARIB | 10 | 0.6 | 0.65 | 136 | 0.252 | 0.261 | 0.113 | 0.374 | 93.7 | 0.367 | 24.7 |
| CALU6-FP6 | VELIPARIB | 10 | 0.6 | 0.518 | 161 | 0.356 | 0.327 | 0.113 | 0.203 | 48 | 0.42 | 17 |
| CALU6-FP6 | VELIPARIB | 10 | 0.61 | 0.595 | 148 | 0.343 | 0.28 | 0.094 | 0.282 | 47.1 | 0.355 | 14.8 |
| CALU6-FP6 | CISPLATIN | 21 | 0.54 | 0.421 | 160 | 0.413 | 0.308 | 0.139 | 0.14 | 94.1 | 0.716 | 38.6 |
| CALU6-FP6 | CISPLATIN | 21 | 0.52 | 0.363 | 174 | 0.453 | 0.329 | 0.145 | 0.073 | 70.2 | 0.748 | 31.6 |
| CALU6-FP6 | CISPLATIN | 21 | 0.53 | 0.346 | 166 | 0.447 | 0.329 | 0.149 | 0.075 | 42.5 | 0.796 | 19 |
| CALU6-FP6 | CISPLATIN | 21 | 0.54 | 0.367 | 171 | 0.472 | 0.35 | 0.104 | 0.074 | 49.3 | 0.658 | 21.7 |
| CALU6-FP6 | CISPLATIN_VELIPARIB | 21 | 0.53 | 0.395 | 160 | 0.441 | 0.336 | 0.137 | 0.085 | 46 | 0.622 | 20.2 |
| CALU6-FP6 | CISPLATIN_VELIPARIB | 21 | 0.53 | 0.456 | 187 | 0.49 | 0.337 | 0.112 | 0.061 | 94.1 | 0.808 | 42.2 |
| CALU6-FP6 | CISPLATIN_VELIPARIB | 21 | 0.53 | 0.349 | 170 | 0.459 | 0.333 | 0.134 | 0.075 | 42.4 | 0.727 | 18.9 |
| CALU6-FP6 | CISPLATIN_VELIPARIB | 21 | 0.55 | 0.442 | 165 | 0.449 | 0.362 | 0.103 | 0.087 | 98.3 | 0.762 | 42.1 |
| CALU6-FP6 | CISPLATIN_VELIPARIB | 21 | 0.54 | 0.416 | 165 | 0.443 | 0.346 | 0.127 | 0.084 | 100.1 | 0.786 | 43.6 |
| CALU6-FP6 | NO TREATMENT | 21 | 0.52 | 0.403 | 174 | 0.485 | 0.348 | 0.111 | 0.056 | 92.6 | 0.848 | 41.9 |
| CALU6-FP6 | NO TREATMENT | 21 | 0.53 | 0.409 | 184 | 0.486 | 0.297 | 0.142 | 0.075 | 75.2 | 0.795 | 34.5 |
| CALU6-FP6 | NO TREATMENT | 21 | 0.54 | 0.41 | 157 | 0.451 | 0.344 | 0.125 | 0.081 | 74.4 | 0.778 | 33 |
| CALU6-FP6 | NO TREATMENT | 21 | 0.53 | 0.434 | 180 | 0.496 | 0.335 | 0.11 | 0.06 | 65.3 | 0.726 | 29.2 |
| CALU6-FP6 | NO TREATMENT | 21 | 0.53 | 0.484 | 169 | 0.484 | 0.322 | 0.132 | 0.062 | 172.9 | 0.847 | 78.1 |
| CALU6-FP6 | VELIPARIB | 21 | 0.58 | 0.773 | 150 | 0.463 | 0.309 | 0.104 | 0.124 | 47.5 | 0.558 | 19.7 |
| CALU6-FP6 | VELIPARIB | 21 | 0.6 | 0.537 | 151 | 0.332 | 0.316 | 0.096 | 0.255 | 45.5 | 0.484 | 15 |
| CALU6-FP6 | VELIPARIB | 21 | 0.55 | 0.475 | 178 | 0.5 | 0.317 | 0.114 | 0.069 | 67.3 | 0.751 | 30 |
| CALU6-FP6 | VELIPARIB | 21 | 0.55 | 0.407 | 165 | 0.451 | 0.337 | 0.124 | 0.088 | 50.6 | 0.764 | 22.1 |
| CALU6-FP6 | VELIPARIB | 21 | 0.56 | 0.724 | 167 | 0.458 | 0.358 | 0.105 | 0.079 | 23.4 | 0.506 | 10 |
| CALU6-FP6 | VELIPARIB | 21 | 0.53 | 0.36 | 171 | 0.438 | 0.334 | 0.145 | 0.084 | 42 | 0.63 | 18.7 |
